# Protective immunity in hamsters from an oral Nipah vaccine correlates with pseudovirus neutralising antibody titre

**DOI:** 10.1038/s41598-026-40205-2

**Published:** 2026-04-02

**Authors:** Meredith Stewart, Peter Bone, Andrew Bacon, Golnaz Emami, Lauren Cave, Elliot J. Bland, Stuart Dowall, Linda Easterbrook, Stephen Findlay-Wilson, Susan Fotheringham, Emma Kennedy, Ines Ruedas-Torres, Francisco J. Salguero, Craig Laferriere, Jeff Drew

**Affiliations:** 1 iosBio Ltd, Hayworthe House, Market Place, Boltro Road, Haywards Heath, RH16 1DB UK; 2 UK Health Security Agency (UKHSA), Porton Down, Salisbury, Wiltshire SP4 0JG UK

**Keywords:** Biotechnology, Diseases, Drug discovery, Immunology, Microbiology

## Abstract

Nipah virus (NiV) is a deadly zoonotic pathogen with a high mortality rate and no approved vaccines, creating an urgent need for new solutions. We developed a novel, thermostable, orally administered NiV vaccine using a human serotype 5 adenovirus (AdHu5) vector to express NiV glycoproteins G and F. This vaccine, delivered via an enteric-coated capsule using the OraPro platform, eliminates the cold chain and allows for self-administration. In a Syrian hamster model, a prime-and-pull strategy—an intramuscular prime followed by an oral booster—provided 100% protection against a lethal NiV challenge, significantly outperforming intramuscular administration alone, which offered only 66% protection. The oral booster also led to higher serum neutralizing antibody titres while reducing anti-vector immunity, potentially enabling repeated use of the same vector. Histopathological analysis showed superior protection in orally boosted animals, with minimal lung lesions. This study highlights the potential of oral vaccines for addressing emerging infectious diseases.

**Supplementary Information:**

The online version contains supplementary material available at 10.1038/s41598-026-40205-2.

## Introduction

Recent outbreaks of Nipah virus (NiV) have underscored the need for effective vaccines^1, 2^. NiV is an enveloped, single-stranded RNA virus within the *Henipavirus* genus of paramyxoviruses^3^. It is a zoonotic pathogen transmitted primarily by the faecal/oral route from fruit bats^4^ and poses a threat to both human and animal health by causing severe illness, including encephalitis and respiratory symptoms, with a mortality rate between 40 and 75% [https://www.who.int/news-room/fact-sheets/detail/nipah-virus].

Despite the significant threat, no commercially available vaccines exist; current treatment options are limited to supportive care and antivirals of restricted utility, though monoclonal antibodies (mAbs) are currently in development^5^. Current vaccine strategies utilise the surface receptor glycoprotein G (and sometimes fusion glycoprotein F) formulated into subunit vaccines^6^, recombinant virus vectors^7–11^, virus-like particles^12,13^, virus replicon particles^14^ and mRNA platforms^15^.

At the UKHSA, we optimised a NiV disease model in hamsters using different challenge routes to understand the variations in model parameters from the different sites^16^. We found that IP administration of the NiV demonstrated a more rapid dissemination to a wider range of tissues and organs than intranasal, though the latter mimicked the natural route of infection through a mucosal surface spreading to the lungs before other organs.

Oral vaccination offers significant advantages over traditional injection-based methods^17^. It can induce mucosal immunity, which is crucial for preventing initial infection, enhance IgA-mediated clearance of infected cells, and reduce viral shedding and onward transmission. Our approach involves a “Prime and Pull” vaccine strategy proposed for a therapeutic herpes simplex 2 vaccine^18,19^, which involved an intramuscular priming dose followed by a chemoattractant to “pull” immune cells to the mucosal tissues. Instead of a chemoattractant, our innovation uses a patented thermostabilised viral vector in an enteric coated capsule^20^ for oral delivery to small intestine where it interacts with the cells of the mucosal immune system. Furthermore, this approach allows the recipient to self-administer the booster dose thus facilitating rapid vaccine distribution and administration in case of an emergency in an outbreak. Previous success with a Zika vaccine demonstrated a robust immune responses and protection in non-human primates from a systemic live Zika challenge^21^.

In this study, we developed a series of human adenovirus type 5 constructs (AdHu5) expressing NiV F and G glycoproteins. The constructs were tested in Syrian hamster models to determine the optimal vaccine candidate. Our findings indicate that Prime and Pull vaccination with AdHu5 provided superior protection compared to intramuscular administration. Notably, the presence of serum neutralising antibodies was correlated with survival, marking the first oral NiV vaccine to demonstrate such efficacy. This research highlights the potential of oral vaccines in addressing emerging infectious diseases, offering a practical solution for controlling NiV outbreaks in both human and animal populations.

## Results

### Preparation and in vitro characterisation of adenovirus 5 vectors with NiV F & G transgenes

**Fig. 1:**
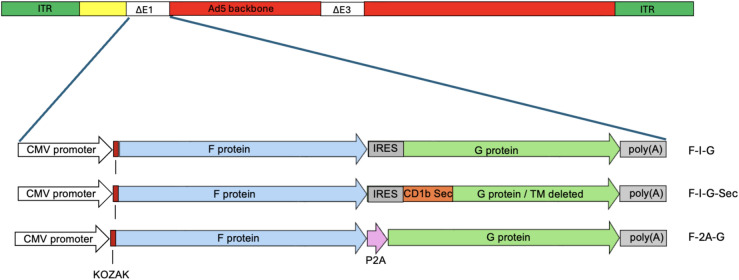
Schematic of the 3 AdHu5 vectors with bicistronic inserts for Nipah F and G glycoproteins. Sequences were separated by an IRES element or a ribosomal skipping 2A sequence. Additionally, one construct harboured a secretory (Sec) motif from CD1b. Labels for each vector are on the right. *ITR* inverted terminal repeat. ∆E1 and ∆E3 indicate deleted genes.

Three human adenovirus 5 (AdHu5) expression cassettes were designed with different genetic elements to co-express the NiV F and NiV G glycoproteins from a single transcript. The human codon optimized F and G transgenes were separated with an internal ribosome entry site (IRES)^22^ or an autocatalytic cleavage site P2A^23^. In the third construct, the transmembrane region of the G protein^24^ was replaced with a secretory signal peptide (Sec)^25^ (Fig. 1). The authenticity and integrity of these constructs were confirmed through full-length sequencing using the Oxford Nanopore Technologies Rapid Sequencing Kit.

**Fig. 2:**
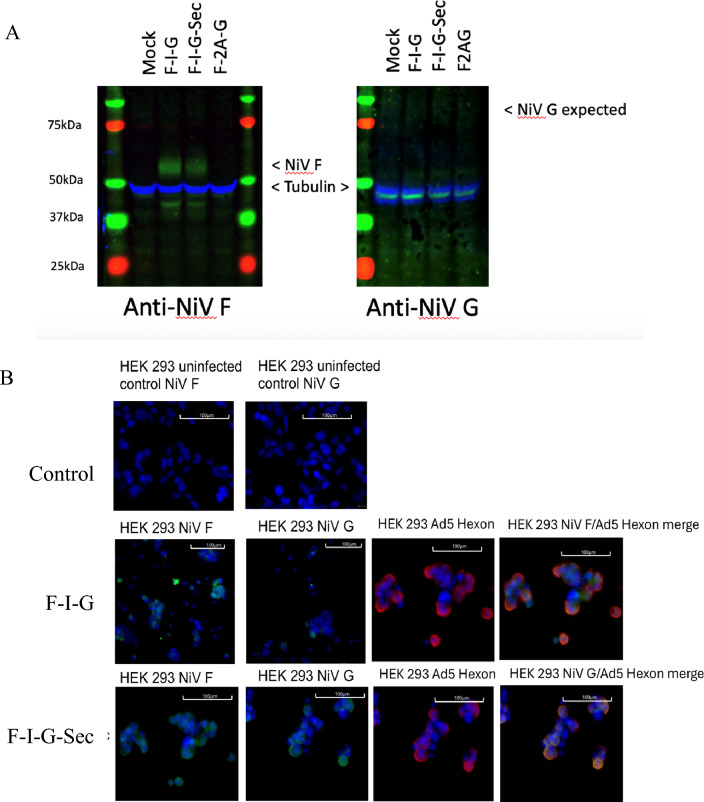
(**A**) Immunoblot of A549 cell lysates after infection with adenoviral constructs indicated. Immunoblots were probed for the presence of the F with MAB12307 and G protein with monoclonal antibody NVG-18. (**B**) Assessment of the presence of F and G proteins expressed from F-I-G and F-I-G-Sec compared to control uninfected cells by immunofluorescence. Blue staining is DAPI. Green staining on the cell surface shows proteins F and G with murine anti-F MAb 13G5 and anti-G MAb NVG-18. Red staining shows adenovirus hexon with rabbit anti-AdHu5 (ab6982). Merged images demonstrated co-expression of adenovirus and F or G antigen. Bar represent size 100 mm.

To confirm the expression of these constructs, HEK293, HuTu-80 and A549 cells were transduced with AdHu5 vectors carrying these cassettes and the presence of the F and G proteins was confirmed by immunoblotting with commercial antibodies. An example with A549 cells is presented in (Fig. 2A). Cells transduced with F-2A-G did not show detectable expression of either F or G glycoproteins, and further results with these constructs are not presented here. Immunoblotting with anti-NiV G MAb12306 detected multiple, unidentified bands, one of which appeared at the expected 75–78 kDa size range (not shown). The immunoblots were repeated with anti-NiV G antibodies 48D3 (not shown) and NVG-18 (Fig. 2A), but no bands were visible aside from tubulin.

Immunofluorescence analysis corroborated some of these findings, revealing no expression of F or G proteins in cells transduced with F-2A-G. However, F-I-G and F-I-G-Sec transduced cells exhibited weak but consistent expression of both F and G proteins, with these proteins colocalising with the AdHu5 as anticipated (Fig. 2B).

### Immunogenicity of candidate vaccines in hamsters and selection for development

**Fig. 3:**
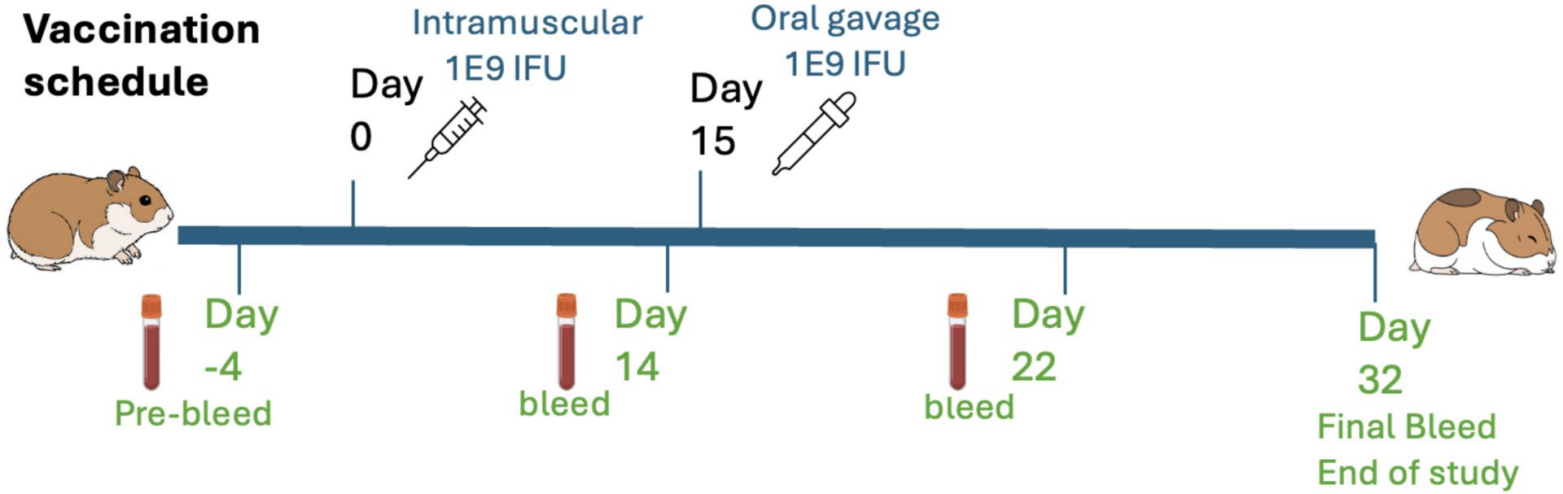
Vaccination schedule and sample collection timeline for study. N = 6 Golden Syrian hamsters per group. The oral gavage was done with liquid vaccine following gavage with bicarbonate to neutralise stomach acid. The time of vaccination and sample harvest are indicated.

To identify which of the constructs was optimal for the AdHu5-NiV vaccine, we performed an immunogenicity study in Syrian hamsters using a rapid screening schedule (Fig. 3). Three groups of 6 hamsters received the constructs F-I-G, F-I-G-Sec and PBS as a negative control. The 1E + 09 IFU/dose was chosen based on previous dose-range studies in rats with the AdHu5 vector.

Serum antibody responses were measured using ELISA coated with a commercial Nipah F protein (ProteoGenix). The conformation of the F protein (pre- or post-fusion) was not described in the datasheet for the product. F-I-G and F-I-G-Sec groups exhibited low antibody levels, with the latter showing marginally higher anti-F IgG titres (Table 1). We tested bronchoalveolar lavage (BAL) and serum for F specific IgA antibodies by ELISA, but the results were negative or below the threshold of detection (starting dilution 1:10, results not shown).

**Table 1:** Immune response in golden syrian hamsters to AdHu5-NiV vectors.

Group	Vaccine route	Anti-F IC_50_ GMT (95%CI)	Anti-G OD_450_ 1:50 pooled sera	F + G pvNAb GMT (95%CI)	G pvNAb GMT (95%CI)
1 (n = 6)	F-I-G-Sec IM/OR	27.96 (16–49)	0.458	76 (38–150)	60 (30–110)
2 (n = 6)	F-I-G IM/OR	26.4 (13–52)	0.371	64 (20–200)	37 (13–110)
3 (n = 4)	PBS	12.5	0.334		

Day 32 (14 days post dose 2) GMT to F antigen measured by ELISA. Anti-G protein IgG OD_450_ measured using pooled sera. F + G and G pseudovirus neutralisation GMT measured by luciferase lentivirus assay with HEK293-T target cells.

*GMT* geometric mean titre, *CI* confidence interval.

An ELISA coated with 0.1 μg/well Nipah G protein (TheAntigenCompany) had a high background with pooled sera from the PBS control group (Table 1). Pooled sera from the F-I-G-Sec group was slightly above this. We concluded that a more specific assay was needed to detect anti-NiV G antibody.

### Development of a pseudovirus neutralisation assay

We developed a pseudovirus neutralisation assay based on published procedures^26,27^ using a 4th generation lentiviral system^28^ with NiV F and G expression plasmids. NiV F contained an 18 aa C′ terminal truncation and NiV G had a 34 aa N′ terminal truncation. We found that pseudovirus with NiV G alone, in the absence of NiV F fusion protein, was able to infect the target HEK-293 T cells. This is similar to observations by Pernet and colleagues who found virus-like particles expressing NiV G were able to infect Chinese hamster ovary (CHO-K1) cells and VeroE6 cells by macropinocytosis^29^. Anti-NiV antibody inhibited this infection, demonstrating its specificity. Since we needed a more sensitive and specific way to measure antibody to NiV G, we decided to measure pseudovirus neutralising antibody (pvNAb) to both NiV F + G and NiV G alone.

pvNAb GMTs for F-I-G and F-I-G-Sec constructs showed similar values with overlapping confidence intervals using both NiV G and NiV F + G pseudoviruses (Table 1). These values are similar to those observed with ChAdOx1 FPG Nipah vaccine in hamsters using a neutralisation assay with NiV on VeroE6 cells^9^. Based on slightly higher values, we selected the F-I-G-Sec construct for further development as AdHu5-NiV.

### Production of the lead NiV vaccine candidate and lyophilisation for improved stability

**Fig. 4:**
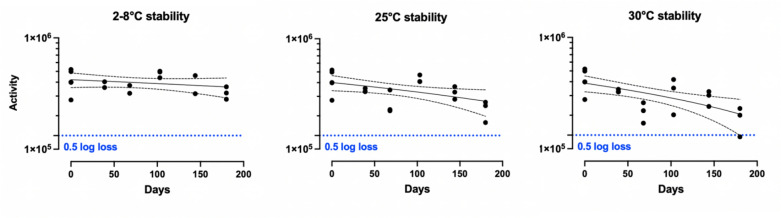
Stability of OraPro-NiV potency. Lyophilised vaccine was stored at (**A**) 2–8 °C (**B**) 25 °C and (**C**) 30 °C for 180 days. Potency was measured by infection of HEK293 cells and expressed as infectious viral particles (IFU) per mg of lyophilised powder. Linear regression was performed using GraphPad. Lines represent 95% CI. Activity declined significantly at 25 and 30 °C (*p* = 0.02 and *p* = 0.003 respectively by linear regression). The dotted blue line represents − 0.5 log loss.

AdHu5-NiVwas amplified and purified by a CDMO (ViraQuest Inc., North Liberty, Iowa, USA). This liquid vaccine was lyophilised using patented methods^20^ and the powder was tested for stability at 2–8 °C, 25 °C and 30 °C for approximately 180 days. In vitro potency, evaluated using Adeno-X™ in HEK293 cells, did not decline over 180 days at 2–8 °C (*p* = 0.28, linear regression) (Fig. 4). Potency declined slightly but significantly at 25 °C and 30 °C (*p* = 0.02 and *p* = 0.003 respectively by linear regression); however, the loss was less than 0.5 log at these temperatures for 180 days.

To prepare OraPro-NiV, freshly lyophilised AdHu5-NiV vaccine was formulated into 9H enteric coated capsules following previously described methods^21^.

### Immunogenicity and efficacy of AdHu5-NiV and OraPro-NiV in Syrian golden hamsters

**Fig. 5:**
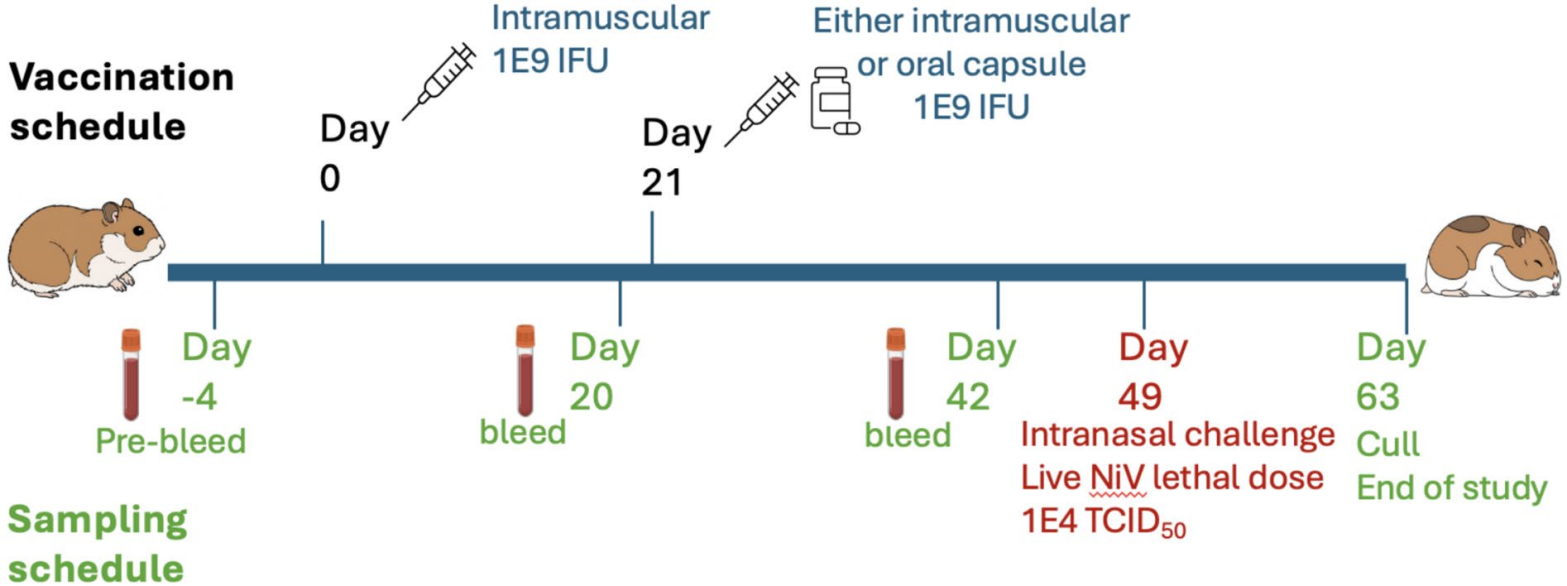
Vaccination of Golden Syrian hamsters with AdHu5-NiV and OraPro-NiV and subsequent challenge with live NiV. Schematic representation of the dosing and sampling schedule. AdHu5-NiV IM doses were prepared from frozen liquid AdHu5-NiV made from F-I-G-Sec. The OraPro-NiV oral capsules were prepared as described in the text.

In collaboration with the Biological Investigations Groups at the UK Health Security Agency, we immunised hamsters according to the schedule in (Fig. 5) to evaluate the oral boosting schedule and its impact on efficacy, immunogenicity, anti-vector immunity and the efficacy of the OraPro-NiV vaccine against live Nipah virus challenge. Details of the schedule are in (Table 2).

**Table 2:** Immunisation regimen with AdHu5-NiV and OraPro-NiV vaccines in golden syrian hamsters.

Group	Number	Prime vaccine	Day	Dose IFU	Route	Booster vaccine	Day	Dose IFU	Route
1.IM/IM	N = 6	AdHu5-NiV	Day 0	1E + 09	IM	AdHu5-NiV	Day 21	1E + 09	IM
2.IM/OR	N = 6	AdHu5-NiV	Day 0	1E + 09	IM	OraPro-NiV	Day 21	9E + 08	Oral
3.PBS	N = 6	PBS	Day 0	Nil	IM	PBS	Day 21	Nil	IM

#### Low antibody response to Nipah F and G proteins

Similar to what was observed in the first experiment, we could not detect a response to NiV G by ELISA. A different NiV G coating protein in this experiment (Acro BioSystems) did not have the high background as seen previously; however, the positive control of MAb 48D3 gave an OD_450_ of only 0.38 at a concentration of 1.4 μg/ml. Only one serum sample gave an OD_450_ above 0.1, and EC_50_ could not be calculated (data not shown). Specificity was improved, but the assay lacked sensitivity.

**Fig. 6:**
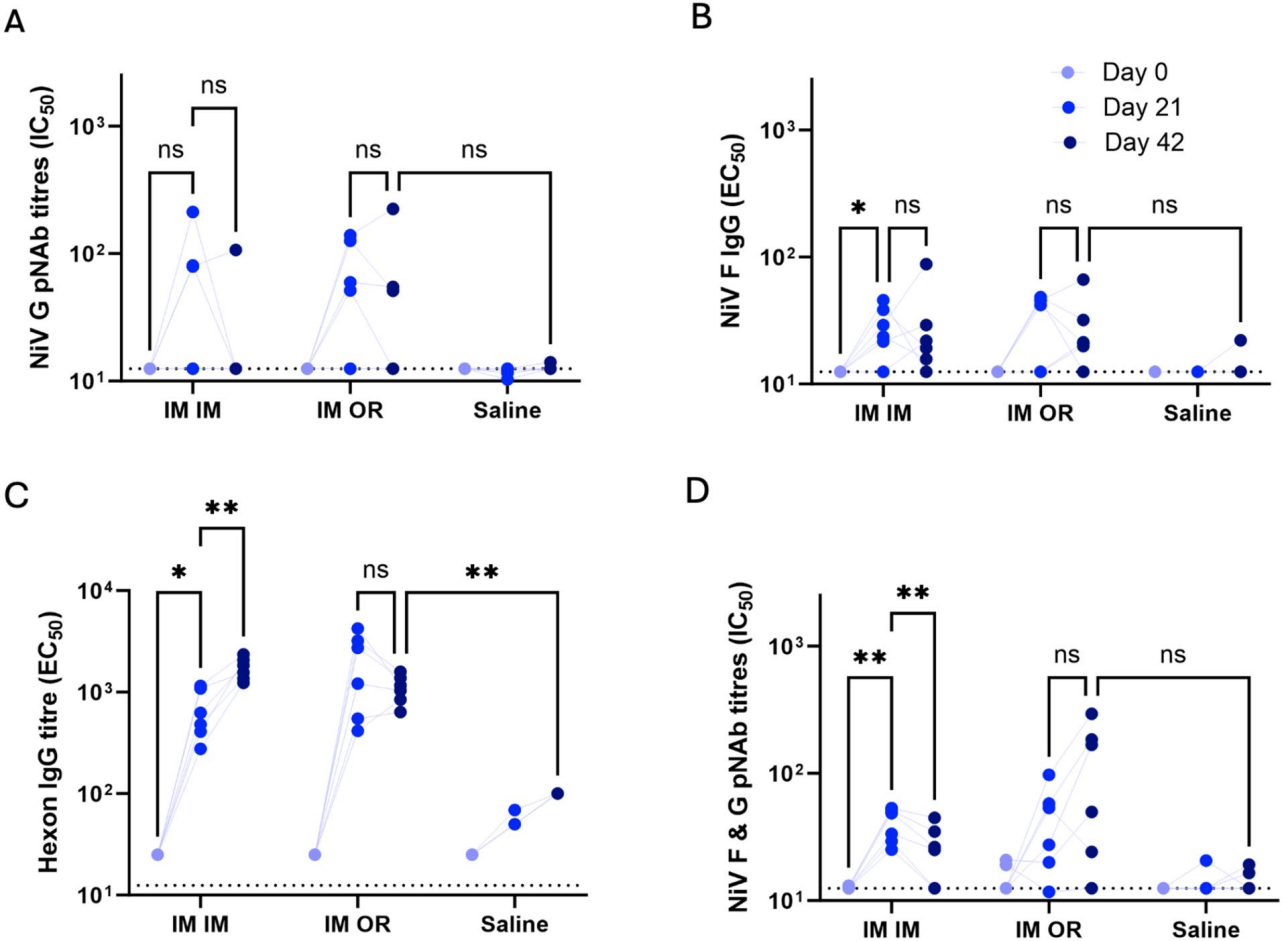
Serum immune responses at pre-bleed, day 21 and day 42 (21 days post boost) in individual Golden Syrian hamsters immunised with AdHu5-NiV and OraPro-NiV. (**A**) pvNAb response to Nipah G only (**B**) IgG response to F protein. (**C**) IgG response to adenovirus hexon protein. The starting dilution on Day 0 was 25, Day 21 was 50 and Day 42 was 100. (**D**) pvNAb response to Nipah F + G. Day 0 (light blue), day 21 (royal blue) and day 42 (dark blue) are shown. ANOVA * = p < 0.05, ** = p < 0.005, ns = not significant.

G only pvNAb titres for individual animals are shown in (Fig. 6A). The IM priming dose, given to both first and second groups, produced 3/6 and 4/6 responders. After boost, only 1 animal in each group showed an increase in G pvNAb at day 42, whereas 2/6 and 3/6 showed a decline in the IM/IM and IM/OR groups respectively. Overall, 5/12 animals never responded. This assay for measuring the response to G appears more sensitive than ELISA, but almost half of the animals did not respond to G.

The IgG responses to the NiV F protein for individual animals as measured by ELISA are shown in (Fig. 6B). 9/12 animals in the combined vaccine groups had responded by day 21. The non-responders boosted after the second dose, but titre declined for the 3 other animals in each group, similar to what was observed for G pvNAb in (Fig. 6A). This cannot be attributed to error in administration, at least for the IM/IM group, because all animals showed a boost to the vector, as shown in (Fig. 6C). Both vaccination regimens induced higher antibody responses against the F protein on day 42 compared to the PBS control (Table 3).

**Table 3:** Serological response to NiV vaccine post-primary, day 21 and post-boost, day 42.

Group route	Anti-F IC_50_ day 21 GMT (95% CI)	Anti-F IC_50_ day 42 GMT (95% CI)	G pvNAb day 21 GMT (95% CI)	G pvNAb day 42 GMT (95% CI)	F + G pvNAb day 21 GMT (95% CI)	F + G pvNAb day 42 GMT (95% CI)
1 IM/IM	26 (18–38)	24 (14–42)	37 (14–100)	18 (9–36)	38 (30–50)	23 (15–36)
2 IM/OR	29 (17–50)	25 (16–39)	45 (19–110)	32 (13–84)	35 (19–66)	72 (26–200)
3 PBS	13 nd	14 (11–17)	12 (11–13)	13 (12–13)	14 (12–16)	14 (12–16)

Samples that had no detectable immune response were given a titre ½ the lowest dilution for statistical calculations.

*nd* not determined, *GMT* geometric mean titre, *CI* confidence interval.

#### Oral administration of the OraPro-NiV induced higher F + G pseudovirus neutralisation titre than intramuscular administration

The F + G pvNAb titres are shown in (Fig. 6D). At day 21 11/12 animals had shown a response, in contrast to only 7/12 seen in the G only assay. Interestingly, while 4/6 animals in the IM/OR group showed an increase in F + G pvNAb titre at day 42, all animals showed a decline in the IM/IM group, and the decline in GMT was significant (GMT ratio = 0.61, *p* = 0.003, Two-Way ANOVA).

The GMTs 21 days after boost are presented in (Table 2). F + G pvNAb titres were higher in both vaccine groups compared to the control. The IM/OR group was similar to the previous result shown in (Table 1) and had higher F + G pvNAb GMT compared to the IM/IM regimen (72 (95%CI 29–193) vs 23 (95% CI 15–36), *p* = 0.18, Two-Way ANOVA).

#### Oral administration of OraPro-NiV did not increase anti-vector immunity

Anti-vector immunity was determined by anti-hexon-IgG levels as measured by ELISA. At baseline, anti-hexon-IgG levels were similar across all groups (Fig. 6C). The PBS placebo showed no response above the limit of detection, which was different for each time point. All animals increased in anti-hexon IgG levels by Day 21. By Day 42, the IM/IM group showed a significant geometric fold increase (GFI) in systemic anti-hexon IgG response (GFI = 2.9, *p* = 0.003 Two-Way ANOVA), whereas the group receiving IM/OR, did not (GFI = 0.72, *p* = 0.3, Two-Way ANOVA).

Altogether these results show that OraPro-NiV administration did not boost the systemic response against the vector while there was a systemic boost against the transgenes. This converse effect aligns with our prior, unpublished findings from studies in rats using AdHu5-based influenza and COVID vaccines. Furthermore, Scanlan and colleagues found similar results in mice with an AdHu5-based influenza vaccine in which IM/IM induced a tenfold higher anti-vector response compared to IM/OR, but a sixfold lower response to the transgene^30^.

### Efficacy of AdHu5-NiV and OraPro-NiV in syrian golden hamsters and correlate of protection

The efficacy of the AdHu5-NiV and OraPro-NiV vaccine in a Syrian hamster challenge model was performed in BSL-4 labs at UKHSA following published procedures^16^. Twenty-eight days post-booster vaccination (Day 49), animals were challenged with 1E + 04 TCID_50_ of live NiV (Malaysian strain; GenBank no. AF212302) via IN administration in a volume of 100 µL per nostril. The animals were monitored twice a day through key disease progression phases to survival or a humane endpoint criterion.

**Fig. 7:**
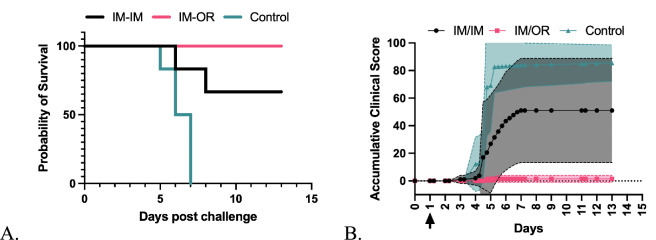
Syrian golden hamsters immunised with NiV vaccine by either IM/IM or IM/OR routes or control. Intranasal challenge was performed with 1E + 04 TCID_50_ of live NiV (Malaysian strain; GenBank no. AF212302) prepared and administered at Porton Down in BSL-4 containment. (**A**) Survival (**B**) Cumulative clinical scores with 95% confidence interval shown as shaded colours.

All animals in the placebo control group started showing clinical signs on day three post challenge and by day five all reached humane end points and were humanely euthanised (Fig. 7A). Of the six animals receiving IM/IM, four survived to the study endpoint and two were euthanised after reaching humane endpoints resulting in significant survival compared to the control group (P = 0.009, Log-Rank survival test). All IM/IM animals showed clinical signs to some degree (Fig. 7B), with the survivors recovering to health by day 7 and remaining healthy until the study endpoint.

All animals survived in the group that received IM/OR (Fig. 7A), so a significant difference compared to the control group (P = 0.001, Log-Rank survival test). Two animals showed minor clinical signs: One animal was breathing rapidly on day five but recovered by day six and remained healthy for the duration of the study (Fig. 7B). One animal had ruffled fur on days five and six, then returned to health until day 11 when it was observed to have ruffled fur and rapid breathing. This animal rapidly returned to good health and remained healthy until the end of the study. Although all animals in the IM/OR route survived, this did not reach statistical significance compared to the IM/IM group (P = 0.138, Log-Rank survival test).

### Histopathological changes and viral RNA distribution in tissues after challenge with NiV

**Fig. 8:**
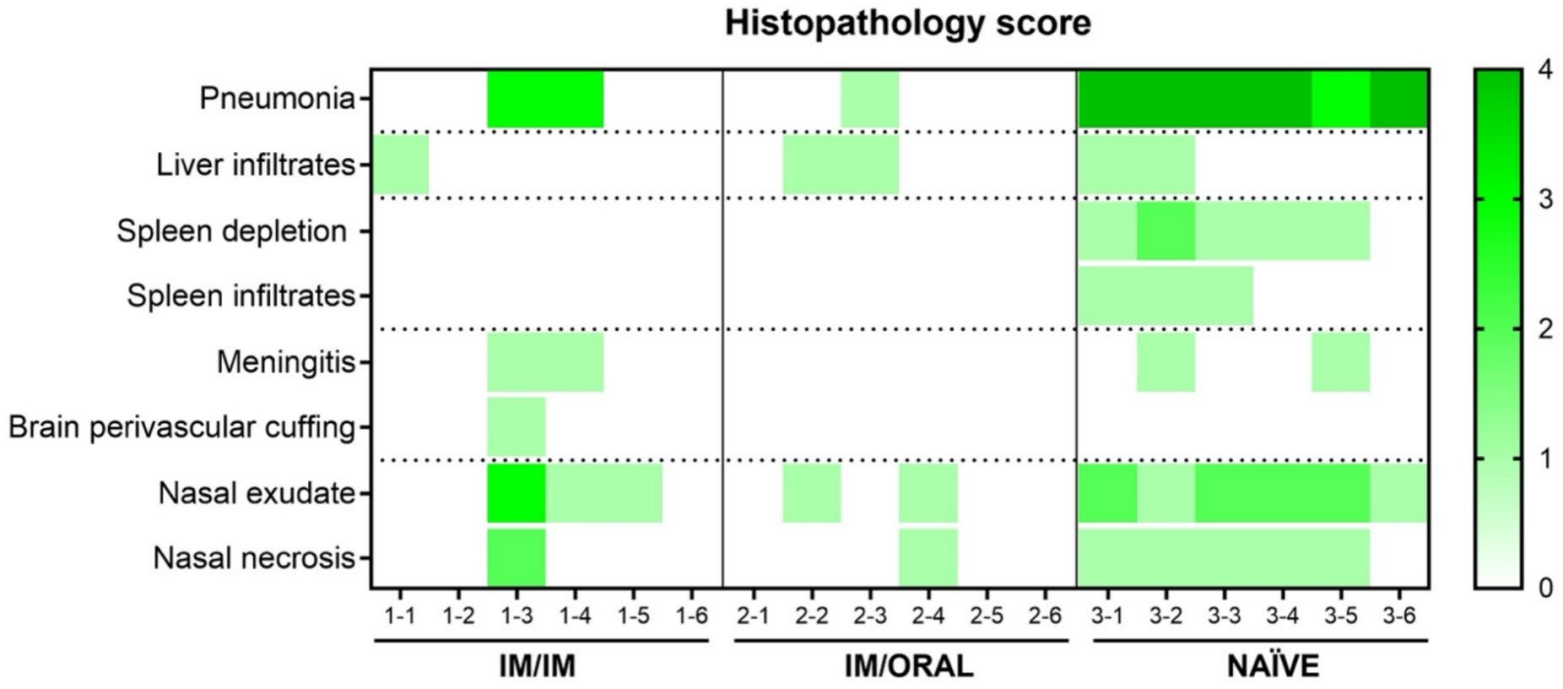
Heatmap of histopathology scores in tissue samples from each animal of different experimental groups. The severity of histopathological lesions in all organs was recorded using a semi-quantitative scoring system. For each parameter, the following scores was applied 0 = within normal limits; 1 = minimal; 2 = mild; 3 = moderate and 4 = marked/severe.

**Fig. 9:**
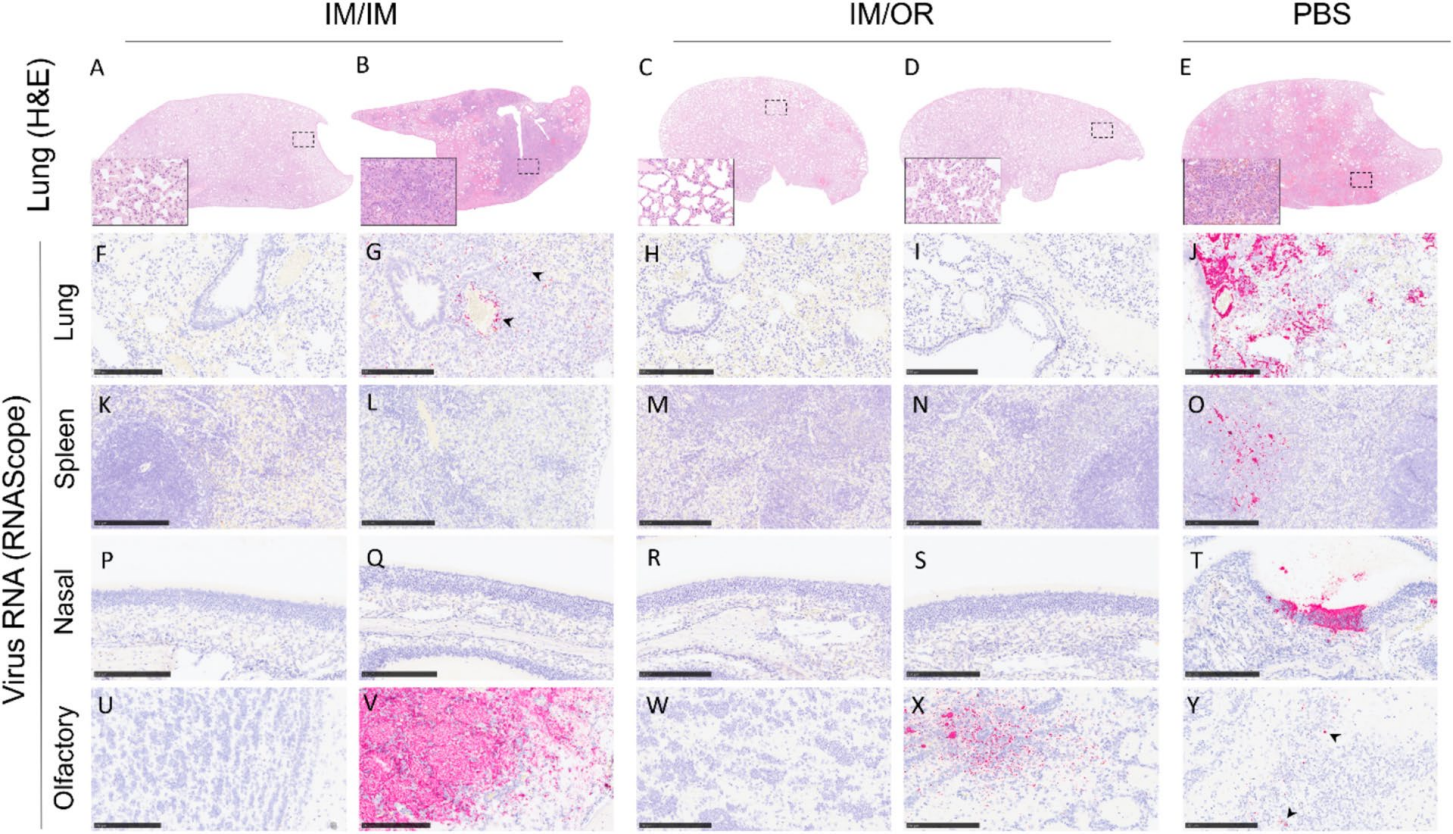
Representative images of lung submacro histopathology (H&E) and RNAScope technique in different organs and experimental groups. (**A**) Lung section without histopathological changes. Inset shows higher magnification. (**B**) Lung section showing moderate broncho-interstitial pneumonia. Inset shows higher magnification of alveolar thickening and pneumocyte type II hyperplasia. (**C**) Lung section without histopathological changes. Inset shows higher magnification. (**D**) Lung section without histopathological changes. Inset shows higher magnification. (**E**) Lung section showing severe broncho-interstitial pneumonia. Inset shows higher magnification of necrosis of alveoli together with heterophils, cell debris, alveolar macrophages and erythrocytes filling the airways. (**F**) Lung section showing no viral RNA. (**G**) Lung section showing viral RNA associated to the areas of broncho-interstitial pneumonia (pink stain, arrowheads). (**H**) Lung section showing no viral RNA. (**I**) Lung section showing no viral RNA. (**J**) Lung section showing viral RNA associated to the areas of broncho-interstitial pneumonia (pink stain). (**K**–**N**) Spleen sections showing no viral RNA. (**O**) Spleen section showing viral RNA within the white pulp (pink stain). (**P**–**S**) Nasal turbinates section showing no viral RNA. (**T**) Nasal turbinates section showing viral RNA within the epithelium (pink stain). (**U**) Olfactory bulb section showing no viral RNA. (**V**) Olfactory bulb section showing of viral RNA (pink stain). (**W**) Olfactory bulb section showing no viral RNA. (**X**) Olfactory bulb section showing viral RNA (pink stain). (**Y**) Olfactory bulb section showing viral RNA (pink stain, arrowheads). Bar = 250 µm.

Histopathology and RNAScope ISH technique were used to compare the type and severity of the lesions, and the presence of viral RNA in tissues from hamsters infected with NiV. Overall, a reduction in the histopathological lesions was observed in the vaccinated groups, being more obvious in tissues from IM/OR group (Fig. 8). All animals from the unvaccinated group (PBS group, 6/6) showed moderate to severe broncho-interstitial pneumonia (Fig. 8), characterized by thickening of the alveolar walls and pneumocyte type II hyperplasia. In the most severe cases, necrosis of alveoli and bronchiolar epithelium was also observed together with heterophils, cell debris, alveolar macrophages, mucus plugs and erythrocytes filling the airways (Fig. 9E, inset). Within vaccinated groups, 2 out of 6 animals from the IM/IM group showed moderate pulmonary lesions (Fig. 9B, inset) and only 1 out of 6 in the IM/OR group showed minimal lesions in the lung, that may be unrelated to the virus infection. Other less remarkable histopathology lesions, mainly present in the PBS group, were mild meningitis, lymphoid depletion in the spleen, inflammatory cell exudates and necrosis of nasal turbinates.

Analysis of RNAScope technique revealed a reduction in the amount of viral RNA in tissues from vaccinated animals, especially in those from IM/OR group where virus was only detected in the olfactory bulb from 3 out of 6 animals (Fig. 9X). In the IM/IM group, apart from the olfactory bulb (Fig. 9V, Supplementary Fig. 3), viral RNA was also detected, in the lung (Fig. 9G, arrowheads) and nasal turbinates from 2 out of 6 animals. However, all animals from unvaccinated group, showed moderate presence of viral RNA in lung (Fig. 9J), spleen (Fig. 9O) and nasal turbinates (Fig. 9T) and 4 out of 6 showed viral RNA in the olfactory bulb (Fig. 9Y, arrowheads) and other areas of the brain.

#### Survival correlated with pseudovirus neutralising antibody titre

**Fig. 10:**
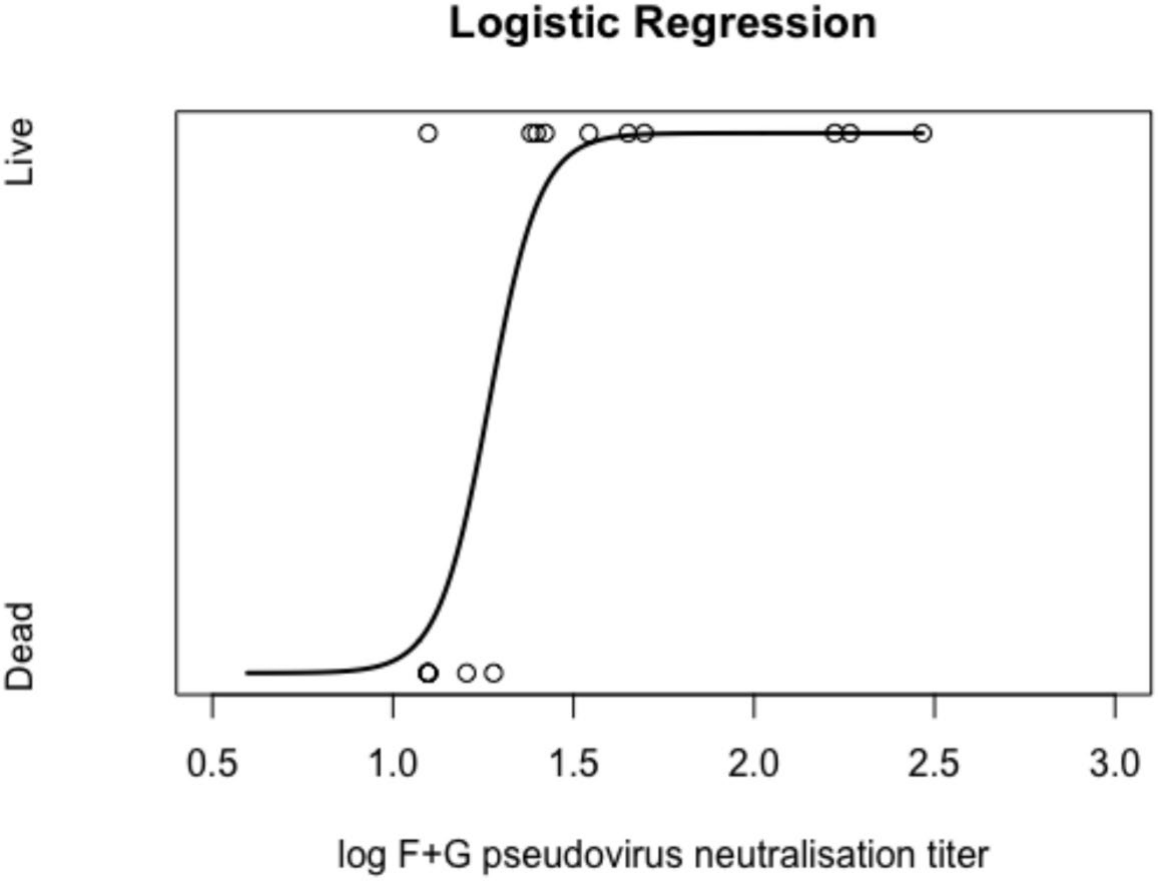
Logistic regression curve of survival vs log F + G pvNAb titre. The regression parameters were: intercept *β*_*0*_ =  − 18.0, *p* = 0.028; Rate parameter *β*_*1*_ = 14.2, *p* = 0.032. The R script used to perform the calculation is in the Supplementary Material.

The correlation between survival and antibody response was estimated by logistic regression using R Studio (code available in Supplementary Material). Survival showed a trend to correlate with log titre F IgG (Supplemental Figure S2A, *p* = 0.07), but not with the log titre G pvNAb (Supplemental Figure S2B, *p* = 0.38). However, log titre F + G pvNAb correlated significantly (Fig. 10, *p* = 0.03). 80% of survivors exhibited titres greater than 1:25, which was the limit of detection in the assay.

## Discussion

This study aimed to design and develop an easily administered, proof-of-concept, oral Nipah vaccine that would provide relevant mucosal immunity against a pathogen transmitted by the faecal-oral route. For this we employed our OraPro platform which utilises lyophilised non-replicating human adenovirus 5 viral vectors with NiV F and G transgenes and formulated into enteric coated capsules for delivery to the small intestine^20^.

The in vitro characterisation of the viral vectors did not provide convincing evidence of the expression of the transgenes when separated by the P2A autocatalytic cleavage site, whereas the vectors with the IRES promoter separating the F and G genes showed evidence of expression, albeit weak, by both immunoblotting and immunofluorescence. This was surprising because P2A was reported to be better than IRES for bicistronic expression^31,32^. We then focussed on the F-I-G and F-I-G-Sec constructs in an immunogenicity study in hamsters with an oral gavage boost in an accelerated immunisation schedule. The resulting antisera were low in IgG to the NiV F protein and had undetectable IgG to NiV G protein by ELISA.

To get a more relevant measure of the immune response, we developed an in-house lentivirus-based neutralisation assay following published methods^27^. During the development of the assay, we observed that pseudovirus with NiV G protein alone was able to infect the target cells. Since we needed a more sensitive and specific method to detect anti-NiV G antibody, we tested all serum samples with the NiV G alone pseudovirus neutralisation assay. This NiV G antibody assay appeared to be more sensitive than the G ELISA, but less sensitive than F + G pseudovirus. We found that F-I-G-sec was slightly more immunogenic. Huang and colleagues also found that the secreted form of the G glycoprotein was most immunogenic^7^. This construct was progressed to the OraPro capsule formulation and challenge study.

We applied the “Prime and Pull” vaccination strategy proposed by the lab of Akiko Iwasaki for a herpes simplex vaccine^19^, except that instead of using a chemoattractant to attract B cells to mucosal tissues^33^ we used OraPro to deliver the vector to the small intestine. This schedule, while having the disadvantage of requiring 2 formats, has additional advantages of stability and ease of administration.

The OraPro formulation employed lyophilisation of the AdHu5-NiV vector^20^ which did not result in a loss of immunogenicity, as seen by comparing the F + G neutralising response to the capsule in (Table 2) (GMT = 76) with the response to the liquid gavage in (Table 1) (GMT = 72). Furthermore, room temperature stability was demonstrated and the loss of in vitro potency at 25 °C and 30 °C was so gradual that the vaccine would still be usable after 180 days. This would be especially useful in the rollout of a mass vaccination campaign in which subjects could receive their first dose by injection and then take the booster dose with them as a capsule. Even if the subject did not have refrigeration, the vaccine potency after 2 to 6 months would be sufficient for the booster dose.

This study included both the fusion (F) and receptor (G) antigens of NiV, hypothesising that presenting multiple antigens simultaneously could have a synergistic effect. Instead we found that the serological neutralisation titres were lower than reported by others for single G antigen adenovirus vectored vaccines^7,8^. This may be due to differences in the assay, immunisation schedule, or low antigen production by the vectors as indicated by the in vitro analysis. Shoji and colleagues^34^ found that immunogenicity of a bicistronic vector was reduced when the genes were joined by an IRES, but increased when joined by 2A. We have observed loss of immunogenicity with other constructs using IRES and 2A, and we are actively searching for alternative methods to make bicistronic vectors.

In the challenge study, 1E + 04 TCID_50_ of live NiV (Malaysian strain) was administered intranasally, modelling the natural route of infection and subsequent clinical symptoms; whereas, many challenge studies report using an IP infection^8,12,13^. Animals receiving placebo vaccinations succumbed to IN challenge within 6 days with severe histopathological damage and high levels of viral RNA, especially in the lungs (Fig. 9). Whilst for initial studies the Malaysian strain of NiV was utilised, further studies to look at protection with other strains (e.g. Bangladesh strain) would generate additional evidence on cross-protection against multiple viral lineages.

We found that the route of vaccine administration made a difference in survival. All animals in the IM/OR group survived, and only 1 animal had minimal lesions in the lungs. In comparison, 4 of the 6 animals in the IM/IM groups developed symptoms, including 2 with severe lesions in the lungs, and they did not survive.

There could be several reasons for the better efficacy of oral administration. We employed the Prime and Pull schedule to stimulate mucosal immunity and provide local protection by generating tissue-resident memory T cells and B cells^19^. To validate this approach, we assayed for F-specific IgA in BAL but the results were negative. This was not surprising considering the low serum antibody concentrations we measured for NiV F and antibody in BAL is transudate from serum rather than locally generated^35^. In future studies, we will also collect saliva, which is more likely to contain antibody synthesized as dimeric IgA by plasma cells in salivary glands and exported by the polymeric Ig receptor^36^. In addition, key immune markers quantified by flow cytometry will be required to detect tissue resident immune memory cells, such as the mucosal homing receptor integrin α_4_β_7_ expressed on intestinal B-cell blasts and plasma cells^37^.

Another explanation for the better protection from oral administration is the higher serum neutralising titres compared to IM boosting. This outcome is particularly noteworthy because, unlike IM injection, oral administration did not boost anti-vector (hexon) titres. These seemingly contradictory findings can be explained by the role of pre-existing immunity. After an IM booster shot, systemic anti-hexon antibodies capture the viral particles; while this boosts the anti-hexon response, it critically inhibits the vector from infecting cells and delivering its transgene. Conversely, the oral route bypasses this problem. Pre-existing systemic immunity is not effectively targeted to the intestine, leaving the vector uninhibited to infect gut cells. This leads to a strong transgene response, driven by the powerful CMV promoter, without provoking a systemic anti-vector response due to the low hexon concentration in the gut.

This has important implications. A clinical study with an HIV vaccine showed that pre-existing immunity to the vector attenuated the response to an injected AdHu5-based vaccine^38^. In our study, it appears that the oral route is less inhibited by systemic anti-vector immunity, allowing further boosting of the antigen using the same vector. This suggests that OraPro holds significant potential as a heterologous booster across a range of vaccines, which could fundamentally reshape and streamline life-cycle immunization programs by offering a versatile and efficient boosting platform.

We found that the F + G pvNAb titres correlated with survival, and the low titres fortuitously enabled us to determine that the correlate of protection was at most a titre of 1:25, the limit of detection in our assay. The G only pvNAbs did not correlate with survival, and this may suggest that the macropinocytosis mechanism of cell entry is minor compared to active fusion. In macropinocytosis, the virus enters the cell in large vesicles that do not undergo the same acidification that smaller endosomal structures experience, allowing the virus to escape into the cytoplasm without the need for low pH to activate its entry^29,39^. Macropinocytosis has also been demonstrated for Respiratory Syncytial Virus (RSV), another member of the *Paramyxoviridae*^40^. The IgG antibody concentration to F antigen weakly correlated with survival. Fusion proteins may exist in 2 conformations, pre- and post-fusion, but this information was lacking for the commercial F protein, and so it is not clear whether the ELISA was measuring relevant antibody. Altogether the data suggest that it is the functional ability of the antibodies that is crucial in virus neutralisation and survival.

Neutralising antibodies are frequently cited as the correlate of protection against viral infections^41^. Escudero-Perez and colleagues made an extensive review of Nipah immune correlates of protection and reported, “it is likely that CoP (for Nipah protection) could be derived from humoral immunity parameters such as numbers of plasmablasts and activated B-cells and specific titers (sic) of IgM and IgG antibodies”^42^. We found that neutralisation titres against a pseudovirus expressing both F + G glycoproteins correlated with survival in the hamster intranasal Nipah challenge model.

Concern has been raised over the safety of injectable adenovirus vaccines, such as those utilising chimpanzee adenovirus (ChAdOx)^43^. Since accidental intravenous injection is often cited as the first step in the initiation of vaccine-induced thrombotic thrombocytopenia (VITT)^44^, we believe that oral administration of non-replicating adenovirus vectored vaccines will not result in entry of the vector into the bloodstream, and thus reduce the chances of adverse systemic events. This study provided supporting data for this position; the second dose with OraPro-NiV selectively boosted anti-NiV responses but did not boost the anti-hexon responses. Furthermore, following the method of Nicolai and colleagues^45^, we performed a study demonstrating that, in contrast to intravenous administration, oral administration of AdHu5 did not induce thrombocytopenia in mice. These results will be reported in full detail shortly.

This study had several limitations. The weak expression of the NiV F and G proteins likely led to the low immunogenicity observed in the hamsters, characterized by low or undetectable IgG antibody levels against the F and G proteins in ELISA tests. Despite this, protection was observed in the Nipah Malaysia challenge model leading to the correlate of protection at the limit of detection of the pseudovirus neutralisation assay. An additional limitation was the inability to definitively prove the mechanism behind the Prime and Pull strategy. We did not include an oral/oral group, which would have helped to unravel the mechanism. While the oral boost provided superior protection, the attempt to measure F-specific IgA in bronchoalveolar lavage (BAL) to confirm a mucosal immune response yielded negative results. Moreover, although animals of IM/OR group survived until the end of the study (day 21) and did not show any clinical sign, a moderate amount of NiV RNA was found in the olfactory bulb by RNAscope (ISH) technique. RNAscope detect viral RNA using specific probes, at the single-cell level with spatial context in tissue sections. However, we have used probes to hybridise with the NiV genome at different target regions, not being able to distinguish between replicating virus vs NiV degraded RNA. Other techniques such as virus isolation were not performed in this study to evaluate and quantify infectious virus in different tissues and organs. The presence of neurological disease should not be excluded if the experiment had been extended to later timepoints. Notably, the occurrence of recrudescent encephalitis is one of the major features of NiV disease in human and occurs after resolution of symptomatic disease^46^. Therefore, long-term follow-up studies would be required to rule out the occurrence of late-onset neurological signs.

Cellular responses are crucial for viral clearance; specifically, CD8 cytotoxic T lymphocytes (CTLs) are capable of identifying and destroying NiV-infected cells by recognizing conserved epitopes, such as those found on the fusion (F) and attachment (G) glycoproteins^47^. Although we did not measure cellular immunity, adenovirus-vectored vaccines offer a promising approach to induce robust cellular immunity against Nipah virus (NiV) by leveraging the inherent ability of these vectors to activate both CD4 and CD8 T cell responses^48^.

In summary, this study showed that OraPro-NiV vaccine, when used as a booster, provided complete protection against NiV infection in Syrian golden hamsters. The findings highlight the potential advantages of the Prime and Pull schedule in generating both systemic and mucosal immune responses, which could be critical for effective prevention and control of NiV infections. Further research will focus on optimising oral administration, improving expression of the transgenes, validating the vaccine’s efficacy against various NiV strains, and exploring mucosal immunity in greater depth.

## Methods

### Cells and culture conditions

HEK-293, HEK-293 T, HuTu-80 and A549 cell lines were obtained from ATCC (catalogue numbers ATCC-CRL-1573.3, ATCC-CRL-3216, ATCC-HTB-40, ATCC-CRM-CCL-185 respectively) and used up to passage 30. Cells were cultured in Dulbecco’s modified essential medium (DMEM, Gibco) supplemented with 10% foetal bovine serum (FBS, Sigma), 50 U/mL of penicillin (Gibco), at 37 °C with 5% CO_2_.

### Vaccine construction

The OraPro-NiV vaccine, designed for oral administration, is based on a non-replicating human adenovirus type 5 (AdHu5) vector, specifically modified by deleting the E1 and E3 regions. Three constructs were engineered to express Nipah virus (NiV) transgenes NiV F and NiV G (GenBank: NC002728). The first construct involved F and G transgenes separated by an encephalomyocarditis virus (EMCV) internal ribosome entry site (IRES)^22^ under the control of a constitutive cytomegalovirus (CMV) promoter (F-I-G). In an alternative version, the transmembrane region of the G protein^24^ was replaced with a secretory signal peptide from human thymocyte antigen CD1a^25^ (F-I-G-Sec) (Fig. 1). The third construct used the autocatalytic cleavage site P2A from porcine teschovirus^23^ to separate the F and G proteins (F-2A-G). The GenBank accession numbers for F-2A-G, F-I-G and F-I-G-Sec are PX246253, PX246254 and PX246255 respectively. These gene cassettes were inserted into the Gateway AdHu5 backbone (ThermoFisher). Briefly, synthetic NiV F and G transgene cassettes in a pMA backbone were manufactured by a CMO (GeneArt, ThermoFisher Scientific, Paisley, UK). PCR amplification was conducted to produce the Kozak-F-IRES-G, Kozak-F-IRES-GSec, and Kozak-F-2A-G cassettes using specific primers with flanking sequences of the pENTR1A plasmid. These cassettes were then ligated into the Gateway shuttle plasmid pENTR1A (ThermoFisher) using the In-Fusion® HD Cloning Kit (Takara Bio). The final vaccine plasmids were prepared by recombining the shuttle vectors into the pAd/CMV/V5-DEST plasmid, resulting in the constructs pF-I-G, pF-I-G-Sec, and pF-2A-G.

### Confirmation of plasmid gene sequences

The authenticity and integrity of these constructs were confirmed through full-length sequencing using the Oxford Nanopore Technologies Rapid Sequencing Kit (SQK-RBK004), following the manufacturer’s protocol. Briefly, 400 ng of the purified plasmid was prepared for sequencing. The DNA was subjected to enzymatic cleavage to generate open-ended molecules, which were subsequently ligated to sequencing adapters containing a motor protein. The prepared library was then loaded onto a MinION R9.4.1 flow cell, and sequencing was initiated via the MinKNOW software.

Real-time base calling and demultiplexing were performed using Guppy, a high-performance base-caller. The resulting reads were aligned to the expected reference plasmid sequence using Minimap2 to assess coverage and identify any discrepancies. A total of 15,000 reads with an average read length of 5.2 kb were obtained, providing an average coverage depth of over 50 × across the entire plasmid. This high coverage ensured reliable detection of single-nucleotide polymorphisms and small indels, confirming > 99.9% sequence identity to the predicted sequence, with no significant structural variations or rearrangements detected.

### Recovery of recombinant AdHu5-expressing NiV antigens

To recover recombinant AdHu5 vectors carrying the NiV antigen cassettes, plasmids were transfected into HEK293 cells. After 96 h post-transfection, the cell culture medium was collected and filtered through a 0.2-µm filter. The cell pellets were then subjected to freeze–thaw cycles in liquid nitrogen to extract the recombinant viruses. These viruses were further amplified in HEK293 cells to generate seed stocks. The viral titre was determined using the Adeno-X Rapid Titre Kit (Takara Adeno-X™) in HEK293 cells. Amplification of the viral stocks were performed by a CDMO (ViraQuest Inc., North Liberty, Iowa, USA).

### Formulation and stability testing of vaccines

Adenovirus-vector vaccines were formulated into thermostable powder and enteric coated capsules following patented methods^20^. Briefly, viral-vector was mixed with a filtered excipient formulation and lyophilised using an optimized cycle. The resulting lyophilized cakes were powdered, sieved and mixed with additives to facilitate capsule filling. Evonik size 9H HPMC L100-55 precoated capsules filled with 1E + 09 IFU of viral vector, sealed, and stored in sealed glass vials.

To evaluate the vaccine’s thermal stability, lyophilised AdHu5-NiV powder was exposed continuously to 3 temperature ranges (2–8 °C, 25 °C and 30 °C) in temperature-controlled chambers. At specific intervals, samples were rehydrated and assessed for infectious units per milligram (IFU/mg) using the Adeno X™ assay.

### Immunoblotting

Whole cell extracts were generated by lysing HEK-293, Hutu-80 and A549 cells previously transduced with F-I-G, F-I-G-Sec or F-2A-G. Equal amounts of protein were resolved by SDS-PAGE and then subjected to immunoblot analysis. Briefly, cells were seeded 24 h prior to infection at 2E + 05 cells/well the media was aspirated and infected with at different MOIs. HEK-293 s were infected with an MOI ~ 1, and Hutu-80, and A549 cells with MOI ~ 100. The MOI is an MOI equivalence-based HEK-293 titre. The virus inoculum was left on the cells for the entirety of the experiment. At different time points post infection, the media was aspirated, cell monolayer harvested in 1 × Laemmli Buffer containing DDT and samples stored at -20 °C.

Prior to electrophoresis, the samples were incubated at 100 °C for 5 min to denature the proteins, and 20 µg of total protein sample was loaded onto a gels NuPAGE 4–12% Bis–Tris polyacrylamide gel alongside a pre-stained protein ladder (BioRAD). Proteins were resolved by SDS-PAGE, in 1 X MES buffer for 2 h at 125 V. Gels were then rinsed in water and equilibrated in ice-cold 1X Tris–Glycine Transfer Buffer with the nitrocellulose membrane (0.2 μm) and filter paper for at least 15 min. The resolved proteins were transferred from the gel to a nitrocellulose membrane in 1 X Tris–Glycine Transfer Buffer (either 10 V overnight or 110 V on ice for 3 h).

For immunodetection of NiV F and NiV G, each blot was first blocked in 5% milk for 1 h at room temperature and then incubated with one of the primary antibodies: rabbit anti-Ad5 (ab6982) and one of the following antibodies: mouse anti-NIPAH F (MBS122198 or Abcam Ab02857-1.1); mouse anti-NIPAH G (MBS122195 or Ab02865-1.1 or MAB12306 or 48D3) for 1 h at room temperature. Membranes were also incubated with rabbit a-tubulin (1:5000 dilution) to monitor loading efficiency. Next, membranes were washed 4 times in 0.1% PBST and then incubated in anti-mouse/ anti-rabbit HRP-labelled secondary antibodies (1:2000 dilution) for 1 h at room temperature. Finally, membranes were washed another 4 times in 0.1% PBST and then incubated in ECL for 1 min prior to scanning with the Azure imaging system.

### Immunofluorescence for the detection of NiV F and G proteins

The day before infection, HEK-293, or A549 or Hutu-80 cells were seeded in a 6-well plate (with a glass coverslip) at a density of 5E + 05 cells per well. 4 h post seeding, cells were infected with F-2A-G, F-I-G and F-I-G-Sec or mock-infected (MOI of 1 for HEK-293 and MOI of 10 for A549 and Hutu-80 cells). Seventy-two hours post infection, media was aspirated, and the cells fixed with 4% (w/v) formaldehyde in PBS. Fixed cells were washed in twice with PBS and then permeabilized with PBS + 0.1% (w/v) Triton X-100. After permeabilising, cells were washed 3 × in PBS to remove any detergent. Cells were blocked with PBS + 5% (v/v) FCS and then incubated with primary antibodies; rabbit anti-Ad5 (ab6982) and one of the following antibodies: mouse anti-NiV F (13G5, MBS122198 or Abcam Ab02857-1.1); mouse anti-NiV G (NVG-18, MBS122195 or Ab02865-1.1) for 1 h at 37 °C. Cells were then washed three times in PBS and further incubated (anti-mouse Alexa Fluor 488 and anti-rabbit Alexa Fluor 647 secondary antibody (1:2000) for 1 h at 37 °C in dark. Cells were washed 3 × in PBS and mounted in Vectashield® mounting medium containing DAPI (4′,6-diamidino-2-phenylindole).

### Animal studies

All procedures were performed in accordance with the United Kingdom Animals (Scientific Procedures) Act 1986. Experiment protocols were approved by ethical review at the UK Health Security Agency by the Animal Welfare and Ethical Review Body (AWERB) (Approval Code: PPL PP3877532). This study is reported in accordance with ARRIVE guidelines. (https://arriveguidelines.org).

Studies with live NiV were conducted as previously described under biosafety level 4 (BCL4) containment at UKHSA with Institutional Biosafety Committee approval^16^.

### Initial immunogenicity study in hamsters with liquid formulations

Immunogenicity studies were conducted to identify the most promising vaccine candidate among three recombinant constructs. Three groups of Syrian golden hamsters (n = 6 per treatment group, n = 4 for the negative PBS control) were used. The animals were inoculated on day 0 with 1 × 10^9^ PFU in 100 µl, either via intramuscular injection (IM) or oral gavage (OR), with an oral booster on day 15. Before oral administration, acid neutralisation was performed using 400 µl of 7.5% sodium bicarbonate. The dosage and use of bicarbonate were chosen based on previous experience with similar AdHu5 vectors in rats. All immunisations were performed under sedation with gaseous Isoflurane (5% O_2_ 4 L/M). Group 1 received F-I-G-Sec IM/OR, Group 2 received F-I-G IM/OR, and Group served as a negative control, receiving PBS.

For sample collecting, the animals were first sedated under Isoflurane (5% O_2_ 4 L/M) and maintained on a mask during bleeding. Serum samples were collected on days -4, 14, 22 and 32.

For cull on day 32, the animals were first sedated under Isoflurane (5% O_2_ 4 L/M) and maintained on a mask during procedures. This was followed by a cardiac overdose of 0.5 ml × 200 mg/ml sodium pentobarbital and tissues were removed after confirmation of cessation of the circulatory system.

Bronchoalveolar lavage (BAL) was collected by the following procedure. The trachea of the hamster was exposed by an incision in the neck. A syringe was used to inject 5 ml of warm PBS gently into the lungs and the fluid was left for 30–60 s. Then the fluid was withdrawn slowly. This was repeated and the samples pooled.

### Live Nipah virus challenge studies

Nipah Virus (NiV) challenge study in hamsters, NIPAH-23–09 (UKHSA study plan 6584) was performed using published procedures^16^. Three groups of Syrian golden hamsters (n = 6 per group were immunised following the schedule in (Table 2). This gave 80% power of seeing 83% protection (5 of 6 animals surviving) when all unimmunised control animals met humane endpoints. Animals were randomised by staff in the animal facility who were blinded to the study design.

NiV Malaysian strain (kindly provided by the Special Pathogens Branch of the Centers for Disease Control and Prevention, Atlanta, USA) was used for the challenge. Stocks were propagated for a single passage on arrival to create a working bank and titrated on VeroE6 cells (European Collection of Cell Cultures, UK). Virus was verified to be absent of mycoplasma by PCR and the sequence confirmed to be as accessioned on GenBank (AF212302).

OraPro-NiV was administered in an enteric capsule described above. All immunisations were performed under sedation with gaseous Isoflurane (5% O_2_ 4 L/M). Serum samples were taken on days -3, 20 and 42. For sample collecting, the animals were first sedated under Isoflurane (5% O_2_ 4 L/M) and maintained on a mask during bleeding. On day 49, 28 days post-boost, animals were challenged with 1E + 04 TCID_50_ of live NiV (Malaysian strain; GenBank no. AF212302) prepared and administered at Porton Down in BSL-4 containment. The virus was delivered via the intranasal route in a volume of 100 µl/nostril. Challenge was given under isoflurane sedation and animals monitored until a full recovery from sedation was observed.

The weight and temperature of the animals was recorded around the same time daily (07:00 to 09:00), the temperature measure using an implantable ID/temperature chip (idENTICHIP with Bio-Thermal, MSD). Clinical signs were initial measured daily and increased with frequency (upto 4 times daily) over the time course of the experiment. The clinical signs were assigned the following score based; 0, healthy; 1, behavioural change, eyes shut, lethagy; 2, ruffled fur; 3, wasp-waisted, arched back, dehydrated; 5, laboured and/or rapid breathing; 8, ataxia; 10, immobility, unsteady gate neurological signs (hypersentivity, light sensitive) and paralysis and 50, for death according to published criteria.

Hamsters were anaesthetised with isoflurane 5% with 4L/min O_2_ maintained on a mask and then given an overdose of 0.5 ml of 200 mg/ml sodium pentobarbital via the cardiac route at the scheduled end of the study (21 days post-challenge) or upon meeting humane clinical endpoint criteria.

### ELISA with NiV F and G protein and adenovirus hexon protein

Polysorp 96-well ELISA plates were coated with 100 µl/well of 1.5 µg/ml NiV F glycoprotein (ProteoGenix PX-P6240-100) or NiV G glycoprotein (NativeAntigen Company REC31637-100, or Acro BioSystems cat. GLM-H52H3) or of 2.5 µg/ml adenovirus hexon protein (The Native Antigen Company; AH01-100). The plates were blocked using PBST0.05 containing 2% (w/v) skimmed milk. For sera, two-fold serial dilutions starting at 1:25 were added and incubated at 37 °C for at least 2 h. For BAL, two-fold serial dilutions starting at 1:10 were added and incubated at 37 °C for at least 2 h. After incubation, the sera or BAL were removed, and the plates were washed three times with PBST0.05. For sera, a secondary HRP-conjugated anti-mouse IgG antibody (Abcam, ab6728) was diluted 1:5000 in PBST0.05 with 2% (w/v) skimmed milk and added to the plates. For BAL, secondary HRP-conjugated anti-hamster IgA antibody (Brookwood Biomedical, SAB3003A) was diluted 1:250 in PBST0.05 with 2% (w/v) skimmed milk and added to the plates. The plates were then incubated for at least 1 h at 37 °C. Following this, the plates were washed three times with PBST0.05, then three times with PBS. The colour signal was developed using TMB (Invitrogen) for 15 min. The enzymatic reaction was stopped by adding 0.4 M sulphuric acid, and the absorbance was measured at 450 nm using an Omega Fluostar plate reader. To calculate EC_50_ values, the unadjusted OD values from the plate reader were imported into GraphPad Prism. The values were normalised for each plate with 0% set to the average of the blank and 100% set to the highest value of the positive control on the plate. EC_50_ was calculated using a ‘log(inhibitor) vs normal response –variable slope’ non-linear regression; the model was constrained to ensure the Hillslope was less than 0.

### Pseudovirus virus neutralisation assays

#### Generation of G and F + G lentivirus

Pseudovirus (or pseudotyped virus) neutralisation assays serve as alternatives to traditional neutralisation assays for viruses requiring high containment (BSL3 & BSL4) by using non-replicating lentivirus pseudovirus particles. The protocol for generating lentiviral particles was adapted from Witting et al.^26^ and Luo et al.^27^, and we used a 4^th^ generation lentiviral system^28^. NiV F and NiV G plasmids were custom-made; NiV F included an 18-amino acid C-terminal truncation, and NiV G had a 34-amino acid N-terminal truncation. The ratio of NiV F to NiV G plasmid concentration was 3:1. HEK293T cells were seeded and allowed to reach 60–80% confluence over 16–24 h before transfection. For each well of a 6-well plate, the following amounts of plasmids were used: 1 µg of the lentivirus backbone, 0.34 µg of NiV G alone, or 0.34 µg of NiV G plus 1.02 µg of NiV F-expressing plasmid, and 0.22 µg each of the Rev, Tat, and Gag/Pol-expressing plasmids, all diluted in OptiMEM. The plasmid mixtures were incubated with 8.8 µl of 0.1% (w/v) PEI for at least 1 h before transfection. Sixteen hours post-transfection, the medium in the wells was changed. The expression of GFP and the formation of syncytia were monitored by light microscopy. Seventy-two to ninety-six hours post-infection, the supernatant was harvested, filtered, and stored at −80 °C. The titre (RFU/ml) was determined in HEK293T cells as described by Crawford et al.^49^ and Ferarra & Temperton^50^.

#### Neutralisation assay

To perform the assay, HEK293T cells were plated in poly-L-lysine treated plates white bottom 96-well plate at 1.1E + 04 cell/well to allow for the cells to adhere. In a sterile 96-well plate, serum was diluted either twofold or threefold from a starting dilution of 1 in 25 in culture media. Pseudovirus were diluted to ~ 1.0E + 05 RFU/well (~ 2E + 06 RFU/ml) and added to the diluted sera and incubated for 1 h then transferred to pre-plated HEK293T cells. The plates were incubated at 37 °C, 5% CO_2_, humidified environment for 68 h. To measure luciferase activity, cell monolayers were washed once with PBS and lysed in passive lysis buffer. The lysed cells were then incubated with 0.5 × luciferase reagent for 5 min before reading on the Omega Fluostar plate reader. The average readings from the infected and uninfected control wells were used to normalise the data. IC_50_ values from four-parameter Hill equation fitting of normalised RFU values were calculated using GraphPad.

### Histopathology

At post-mortem, tissues from PBS and vaccinated groups were collected, fixed in 10% neutral-buffered formalin, embedded in paraffin, and stained with haematoxylin and eosin (H&E). Slides were digitalised using a Hamamatsu S360 digital slide scanner (Hamamatsu Photonics K.K., Shizuoka, Japan) and examined with the ndp.view2 software (Hamamatsu Photonics K.K., v2.8.24). Tissue evaluation was performed in a blinded manner to avoid subjective bias. The severity of histopathological lesions in all organs was recorded using a semi-quantitative scoring system. Briefly, lung tissues were assessed for the severity of broncho-interstitial pneumonia, liver tissues for the presence of infiltrates, spleen tissues for the presence of inflammatory infiltrates and depletion, and brain tissues were checked for meningitis and perivascular cuffing. In nasal turbinates the presence of inflammatory exudates and necrosis of the epithelium was assessed as well. the For each parameter, the following scores was applied 0 = within normal limits; 1 = minimal; 2 = mild; 3 = moderate and 4 = marked/severe.

### RNAScope in situ hybridization technique

Four µm sections were submitted to RNAScope in situ hybridisation (ISH) technique to detect NiV RNA. The technique was performed automatically on the Leica BOND-RX (Leica Microsystems, Milton Keynes, United Kingdom). This involved pre-treating slides with hydrogen peroxide, performing antigen retrieval for 15 min at 98–101 °C, and incubating with protease plus for 30 min at 40 °C (Advanced Cell Diagnostics, CA, USA). A NiV-specific probe (Cat No. 439258, Advanced Cell Diagnostics) was then applied to the tissues at 40 °C for 2 h. Signal was amplified by using the RNAScope 2.5 HD Detection Kit – RED (Advanced Cell Diagnostics). RNAscope stained slides were mounted using EcoMount (Biocare Medical, CA, USA). Digital images were captured with a Hamamatsu S360 slide scanner and analysed using ndp.view2 software. Nikon NIS-Ar software (Nikon, Praha, Czech Republic) was employed for digital image analysis to quantify the stained area in each tissue sections.

Histopathological and RNAScope ISH techniques were carried out in a ISO9001:2015 and GLP compliant laboratory.

### Statistical analysis

Statistical analysis was performed using GraphPad Prism 10.2.3. Specific analyses for each dataset are detailed in the figure captions. Thermostability IFU assays were analysed by linear regression. Survival comparisons were assessed using non-parametric distribution analysis with Kaplan–Meier plots and right censoring. The Mann–Whitney U test was used to determine significance between groups, with a significance level of ≤ 0.05. Two-Way ANOVA was performed with GraphPad. Logistic regression modelling to examine the association between neutralising antibodies and outcomes was conducted using R.

## Supplementary Information

Supplementary Information.

## Data Availability

Data presented in this study are described in the Supplementary Materials, or available upon request. Nucleotide sequences are available at GenBank accession numbers PX246253, PX246254 and PX246255 at https://www.ncbi.nlm.nih.gov/genbank/.

## Code availability

R code used for logistic regression is available in the Supplementary Materials.

## References

[1] Soni, M., Kumar, V., Singh, M. P., Shabil, M. & Sah, S. Nipah virus resurgence: a call for preparedness across states. *Infect. Med.* **3** (4), 100145 (2024). 10.1016/j.imj.2024.100145 PMC11609362 39624058

[2] Olatunji, G., Kokori, E., Abdulrahmon, M. A. & Aderinto, N. Addressing the recurrent Nipah Virus outbreaks: A call for vigilance, collaboration, and preparedness. *New Microb. New Infect.* **54**, 101184 (2023). 10.1016/j.nmni.2023.101184 PMC10522897 37772170

[3] Gazal, S. et al. Nipah and hendra viruses: Deadly zoonotic paramyxoviruses with the potential to cause the next pandemic. *Pathog. Basel Switz.* **11** (12), 1419 (2022). 10.3390/pathogens11121419 PMC9784551 36558753

[4] Singh, R. K. et al. Nipah virus: epidemiology, pathology, immunobiology and advances in diagnosis, vaccine designing and control strategies - a comprehensive review. *Vet. Q.* **39** (1), 26–55 (2019). 31006350 10.1080/01652176.2019.1580827 PMC6830995

[5] Playford, E. G. et al. Safety, tolerability, pharmacokinetics, and immunogenicity of a human monoclonal antibody targeting the G glycoprotein of henipaviruses in healthy adults: a first-in-human, randomised, controlled, phase 1 study. *Lancet Infect. Dis.* **20** (4), 445–454 (2020). 32027842 10.1016/S1473-3099(19)30634-6

[6] Moon, S. Y. et al. Immunogenicity and neutralization of recombinant vaccine candidates expressing F and G glycoproteins against Nipah virus. *Vaccines* **12** (9), 999 (2024). 39340029 10.3390/vaccines12090999 PMC11436239

[7] Huang, X. et al. Nipah virus attachment glycoprotein ectodomain delivered by type 5 adenovirus vector elicits broad immune response against NiV and HeV. *Front. Cell Infect. Microbiol.*10.3389/fcimb.2023.1180344/full (2023). 37577376 10.3389/fcimb.2023.1180344 PMC10413271

[8] Lu, M. et al. Both chimpanzee adenovirus-vectored and DNA vaccines induced long-term immunity against Nipah virus infection. *NPJ Vaccines* **8** (1), 1–12 (2023). 37925490 10.1038/s41541-023-00762-3 PMC10625554

[9] van Doremalen, N. et al. A single-dose ChAdOx1-vectored vaccine provides complete protection against Nipah Bangladesh and Malaysia in Syrian golden hamsters. *PLoS Negl. Trop. Dis.* **13** (6), e0007462 (2019). 31170144 10.1371/journal.pntd.0007462 PMC6581282

[10] Lu, M. et al. Single-dose intranasal AdC68-vectored vaccines rapidly protect Syrian hamsters against lethal Nipah virus infection. *Mol. Ther. J. Am. Soc. Gene Ther.* **33** (7), 3270–3285 (2025). 10.1016/j.ymthe.2025.03.032 PMC12266052 40143544

[11] Woolsey, C. et al. Recombinant vesicular stomatitis virus-vectored vaccine induces long-lasting immunity against Nipah virus disease. *J. Clin. Invest.* **133** (3), e164946 (2023). 36445779 10.1172/JCI164946 PMC9888376

[12] Chen, S. et al. Ferritin nanoparticle-based Nipah virus glycoprotein vaccines elicit potent protective immune responses in mice and hamsters. *Virol. Sin.* **S1995-802X** (24), 00144–00145 (2024). 10.1016/j.virs.2024.09.005 PMC11738763 39293542

[13] Zhou, D. et al. An attachment glycoprotein nanoparticle elicits broadly neutralizing antibodies and protects against lethal Nipah virus infection. *NPJ Vaccines* **9** (1), 158 (2024). 39217188 10.1038/s41541-024-00954-5 PMC11365981

[14] Welch, S. R. et al. Single-dose mucosal replicon-particle vaccine protects against lethal Nipah virus infection up to 3 days after vaccination. *Sci. Adv.* **9** (31), eadh4057 (2023). 37540755 10.1126/sciadv.adh4057 PMC10403222

[15] Pedrera, M. et al. Evaluation of the immunogenicity of an mRNA vectored Nipah virus vaccine candidate in pigs. *Front. Immunol.* **15**, 1384417 (2024). 38726013 10.3389/fimmu.2024.1384417 PMC11079202

[16] Findlay-Wilson, S. et al. Establishment of a Nipah virus disease model in hamsters, including a comparison of intranasal and intraperitoneal routes of challenge. *Pathog. Basel Switz.* **12** (8), 976 (2023). 10.3390/pathogens12080976 PMC10458503 37623936

[17] Vela Ramirez, J. E., Sharpe, L. A. & Peppas, N. A. Current state and challenges in developing oral vaccines. *Adv. Drug Deliv. Rev.* **114**, 116–131 (2017). 28438674 10.1016/j.addr.2017.04.008 PMC6132247

[18] Bernstein, D. I. et al. Successful application of prime and pull strategy for a therapeutic HSV vaccine. *NPJ Vaccines.* **4** (1), 1–10 (2019). 31396405 10.1038/s41541-019-0129-1 PMC6671986

[19] Shin, H. & Iwasaki, A. A vaccine strategy that protects against genital herpes by establishing local memory T cells. *Nature* **491** (7424), 463–467 (2012). 23075848 10.1038/nature11522 PMC3499630

[20] Drew J. Virus. US12016949B2, https://patents.google.com/patent/US12016949B2/en?oq=Drew+OraPro+US12016949 (2024).

[21] Bacon, A. et al. Generation of a thermostable, oral Zika vaccine that protects against virus challenge in non-human primates. *Vaccine* **S0264-410X** (23), 00198 (2023). 10.1016/j.vaccine.2023.02.055 36894395

[22] Bochkov, Y. A. & Palmenberg, A. C. Translational efficiency of EMCV IRES in bicistronic vectors is dependent upon IRES sequence and gene location. *BioTechniq.* **41** (3), 283–284 (2006). 10.2144/000112243 16989088

[23] Kim, J. H. et al. High cleavage efficiency of a 2A peptide derived from porcine teschovirus-1 in human cell lines, zebrafish and mice. *PLoS ONE* **6** (4), e18556 (2011). 21602908 10.1371/journal.pone.0018556 PMC3084703

[24] Bossart, K. N. et al. Receptor binding, fusion inhibition, and induction of cross-reactive neutralizing antibodies by a soluble G glycoprotein of Hendra virus. *J. Virol.* **79** (11), 6690–6702 (2005). 15890907 10.1128/JVI.79.11.6690-6702.2005 PMC1112112

[25] Martin, L. H., Calabi, F., Lefebvre, F. A., Bilsland, C. A. & Milstein, C. Structure and expression of the human thymocyte antigens CD1a, CD1b, and CD1c. *Proc. Natl. Acad. Sci.* **84** (24), 9189–9193 (1987). 2447586 10.1073/pnas.84.24.9189 PMC299718

[26] Witting, S. R., Vallanda, P. & Gamble, A. L. Characterization of a third generation lentiviral vector pseudotyped with Nipah virus envelope proteins for endothelial cell transduction. *Gene Ther.* **20** (10), 997–1005 (2013). 23698741 10.1038/gt.2013.23 PMC3839624

[27] Luo, X. et al. Establishment of a neutralization assay for Nipah virus using a high-titer pseudovirus system. *Biotechnol. Lett.* **45** (4), 489–498 (2023). 36680637 10.1007/s10529-023-03351-5 PMC9860241

[28] Berkhout, B. A fourth generation lentiviral vector: Simplifying genomic gymnastics. *Mol. Ther.* **25** (8), 1741–1743 (2017). 28772133 10.1016/j.ymthe.2017.06.005 PMC5542795

[29] Pernet, O., Pohl, C., Ainouze, M., Kweder, H. & Buckland, R. Nipah virus entry can occur by macropinocytosis. *Virology* **395** (2), 298–311 (2009). 19854459 10.1016/j.virol.2009.09.016

[30] Scallan, C. D., Tingley, D. W., Lindbloom, J. D., Toomey, J. S. & Tucker, S. N. An adenovirus-based vaccine with a double-stranded RNA adjuvant protects mice and ferrets against H5N1 avian influenza in oral delivery models. *Clin. Vaccine Immunol. CVI.* **20** (1), 85–94 (2013). 23155123 10.1128/CVI.00552-12 PMC3535765

[31] Chan, H. Y. et al. Comparison of IRES and F2A-based locus-specific multicistronic expression in stable mouse lines. *PLoS ONE* **6** (12), e28885 (2011). 22216134 10.1371/journal.pone.0028885 PMC3244433

[32] Kuzmich, A. I., Vvedenskii, A. V., Kopantzev, E. P. & Vinogradova, T. V. Quantitative comparison of gene co-expression in a bicistronic vector harboring IRES or coding sequence of porcine teschovirus 2A peptide. *Russ. J. Bioorg. Chem.* **39** (4), 406–416 (2013). 10.1134/s1068162013040122 24707727

[33] Streeter, P. R., Berg, E. L., Rouse, B. T., Bargatze, R. F. & Butcher, E. C. A tissue-specific endothelial cell molecule involved in lymphocyte homing. *Nature* **331** (6151), 41–46 (1988). 3340147 10.1038/331041a0

[34] Shoji, M., Yoshizaki, S., Mizuguchi, H., Okuda, K. & Shimada, M. Immunogenic comparison of chimeric adenovirus 5/35 vector carrying optimized human immunodeficiency virus clade C genes and various promoters. *PLoS ONE* **7** (1), e30302 (2012). 22276174 10.1371/journal.pone.0030302 PMC3261887

[35] Forrest, B. D., LaBrooy, J. T., Robinson, P., Dearlove, C. E. & Shearman, D. J. Specific immune response in the human respiratory tract following oral immunization with live typhoid vaccine. *Infect. Immun.* **59** (3), 1206–1209 (1991). 1997425 10.1128/iai.59.3.1206-1209.1991 PMC258392

[36] Brandtzaeg, P. Do salivary antibodies reliably reflect both mucosal and systemic immunity?. *Ann. N. Y. Acad. Sci.* **1098**, 288–311 (2007). 17435136 10.1196/annals.1384.012

[37] Farstad, I. N. et al. Human intestinal B-cell blasts and plasma cells express the mucosal homing receptor integrin alpha 4 beta 7. *Scand. J. Immunol.* **42** (6), 662–672 (1995). 8552990 10.1111/j.1365-3083.1995.tb03709.x

[38] Zak, D. E. et al. Merck Ad5/HIV induces broad innate immune activation that predicts CD8^+^ T-cell responses but is attenuated by preexisting Ad5 immunity. *Proc. Natl. Acad. Sci. USA* **109** (50), E3503-3512 (2012). 23151505 10.1073/pnas.1208972109 PMC3528489

[39] Chang, Y. Y., Enninga, J. & Stévenin, V. New methods to decrypt emerging macropinosome functions during the host-pathogen crosstalk. *Cell Microbiol.* **23** (7), e13342 (2021). 33848057 10.1111/cmi.13342 PMC8365644

[40] Krzyzaniak, M. A., Zumstein, M. T., Gerez, J. A., Picotti, P. & Helenius, A. Host cell entry of respiratory syncytial virus involves macropinocytosis followed by proteolytic activation of the F protein. *PLoS Pathog.* **9** (4), e1003309 (2013). 23593008 10.1371/journal.ppat.1003309 PMC3623752

[41] Plotkin, S. A. Recent updates on correlates of vaccine-induced protection. *Front. Immunol.*10.3389/fimmu.2022.1081107 (2023). 36776392 10.3389/fimmu.2022.1081107 PMC9912984

[42] Escudero-Pérez, B., Lawrence, P. & Castillo-Olivares, J. Immune correlates of protection for SARS-CoV-2, Ebola and Nipah virus infection. *Front. Immunol.* **14**, 1156758 (2023). 37153606 10.3389/fimmu.2023.1156758 PMC10158532

[43] Travieso, T., Li, J., Mahesh, S., Mello, J. D. F. R. E. & Blasi, M. The use of viral vectors in vaccine development. *NPJ Vaccines* **7** (1), 75 (2022). 35787629 10.1038/s41541-022-00503-y PMC9253346

[44] Abrignani, M. G. et al. COVID-19, vaccines, and thrombotic events: A narrative review. *J. Clin. Med.* **11** (4), 948 (2022). 35207220 10.3390/jcm11040948 PMC8880092

[45] Nicolai, L. et al. Thrombocytopenia and splenic platelet-directed immune responses after IV ChAdOx1 nCov-19 administration. *Blood* **140** (5), 478–490 (2022). 35486845 10.1182/blood.2021014712 PMC9060731

[46] Sejvar, J. J. et al. Long-term neurological and functional outcome in Nipah virus infection. *Ann. Neurol.* **62** (3), 235–242 (2007). 17696217 10.1002/ana.21178

[47] Liew, Y. J. M. et al. The immunobiology of Nipah virus. *Microorganisms* **10** (6), 1162 (2022). 35744680 10.3390/microorganisms10061162 PMC9228579

[48] Afkhami, S., Yao, Y. & Xing, Z. Methods and clinical development of adenovirus-vectored vaccines against mucosal pathogens. *Mol. Ther. Methods Clin. Dev.* **3**, 16030 (2016). 27162933 10.1038/mtm.2016.30 PMC4847555

[49] Crawford, K. H. D. et al. Protocol and reagents for pseudotyping lentiviral particles with SARS-CoV-2 spike protein for neutralization assays. *Viruses* **12** (5), 513 (2020). 32384820 10.3390/v12050513 PMC7291041

[50] Ferrara, F. & Temperton, N. Pseudotype neutralization assays: From laboratory bench to data analysis. *Methods Protoc.* **1** (1), 8 (2018). 31164554 10.3390/mps1010008 PMC6526431

