# Supplementary material for: Protective immunity in hamsters from an oral Nipah vaccine correlates with pseudovirus neutralising antibody titre
Source: Sci Rep. 2026 Apr 2;16:15763. doi: 10.1038/s41598-026-40205-2 (PMC13194880; doi:10.1038/s41598-026-40205-2)
Supplement: Supplementary file 1 — Supplementary Information. [file 41598_2026_40205_MOESM1_ESM.docx]

SUPPLEMENTARY MATERIALS

# Supplementary Figure 1

Detection of anti-hexon IgG response in the vaccinated groups. (A) Change in anti hexon IgG IC50 with time. The titres for day 0 (post vaccination), day 14 and day 33 are shown. (B) Individual response of each hamster within a group. The geometric mean and 95% confidence intervals are shown. Dotted line shows the limit of detection.


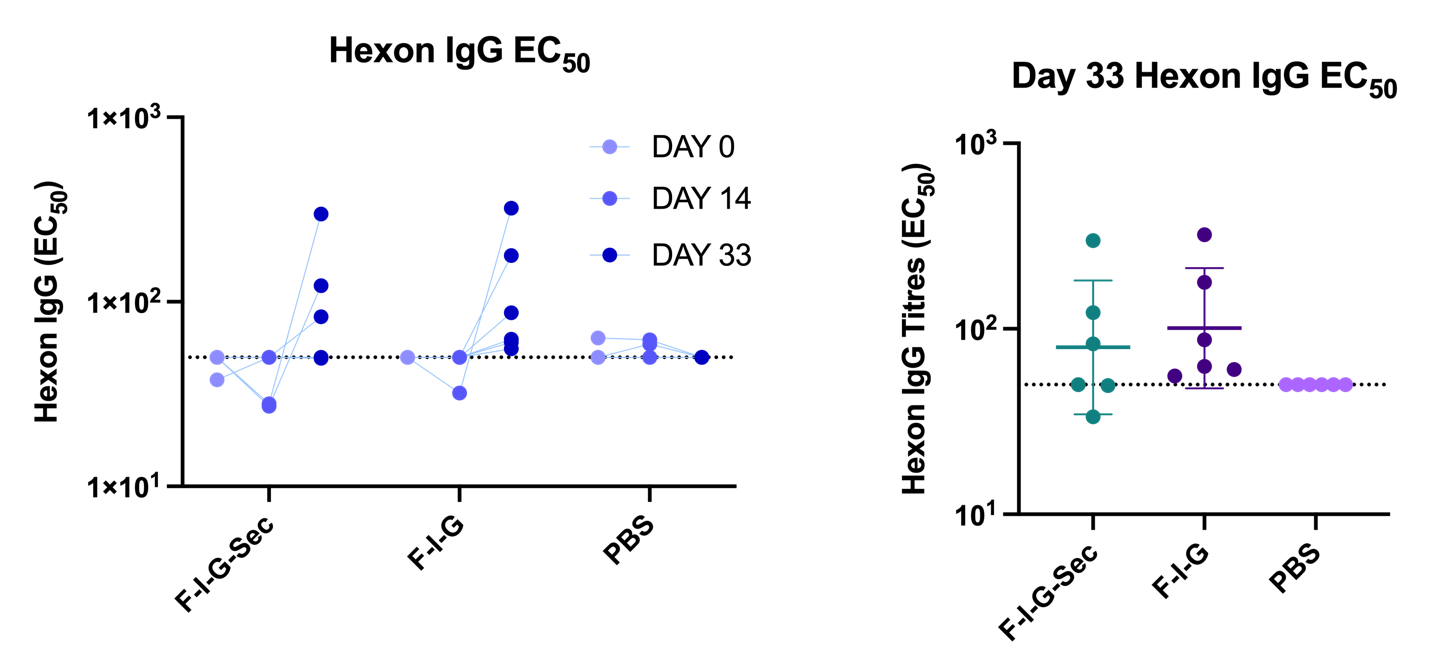


# Supplementary Figure 2

Logistic regression curves calculated for survival versus A) F ELISA IgG titre and B) log G pvNAb titres. Data used for the calculations are listed in S7.

A.


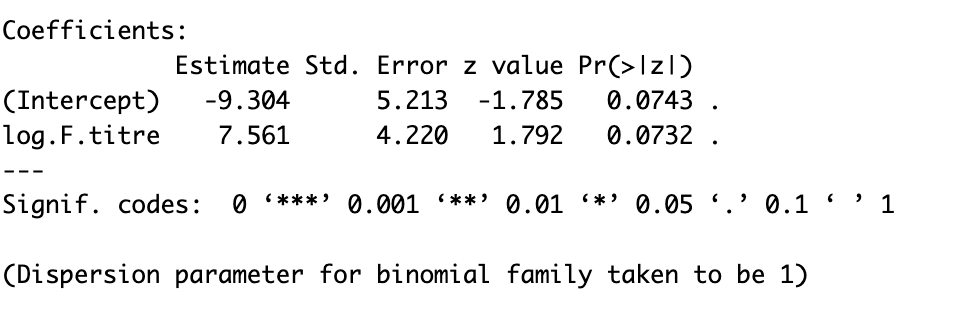

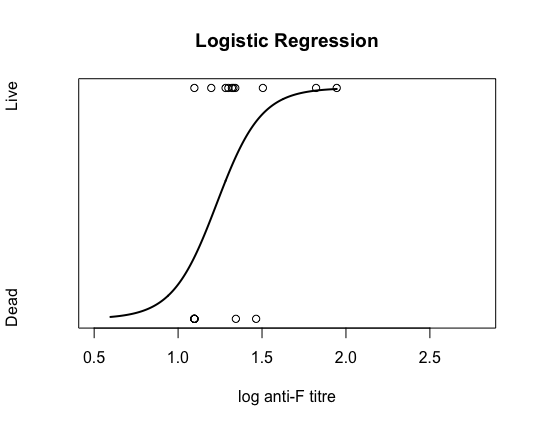


B.


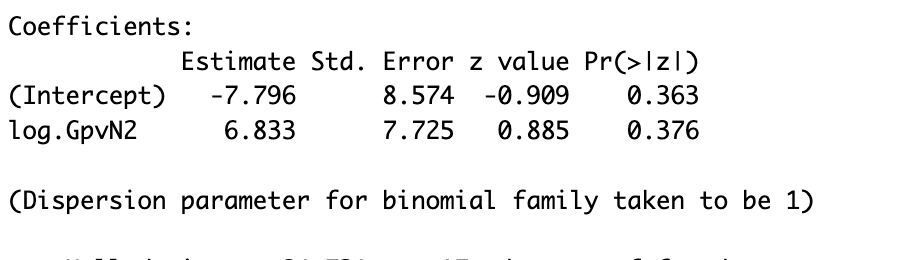

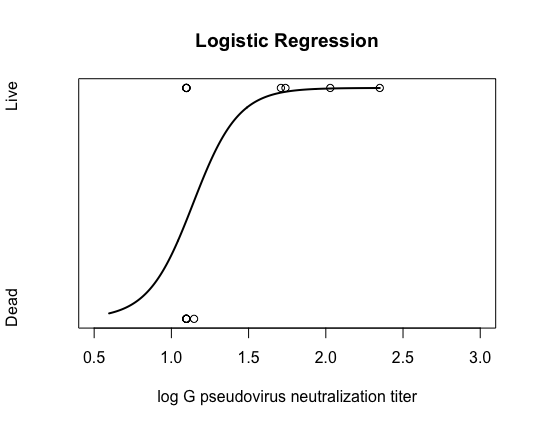


**Supplementary Figure 3**

H&E image corresponding to the matched ISH field showed in Figure 9X (olfactory bulb from an IM/OR animal). Mild gliosis was observed in association with NiV RNA–positive region. Scale bar 100µm.


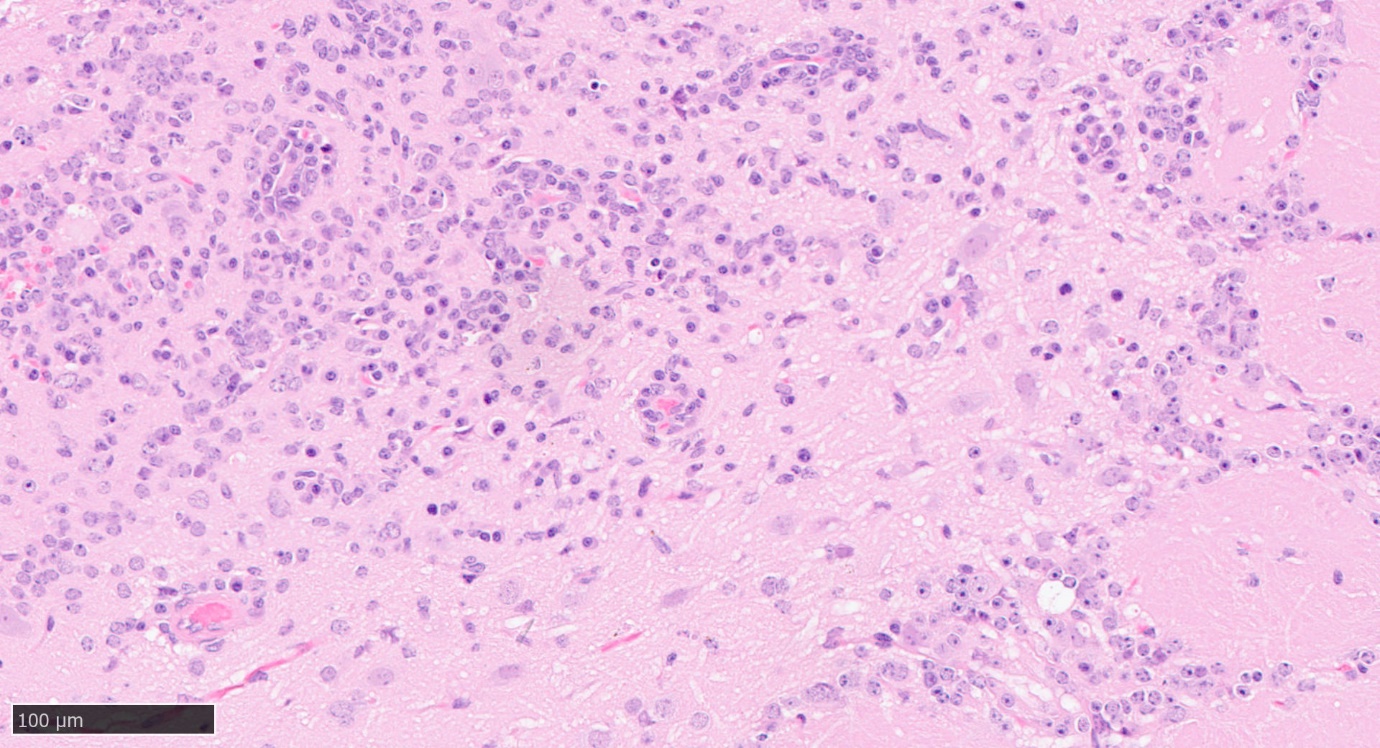


# Supplementary Table 1

Antibodies

| Antibody | Vendor | Cat number | Dilution |
| --- | --- | --- | --- |
| anti-Nipah virus F1 (clone13G5) | Szabo Scandic or Absolute Antibody | ABAAb02857-23.3 or  MBS122198 | 1:2000 |
| anti-Nipah virus F1 MAB12307 | The Native Antigen Company | MAB12307-500 | 1:2000 |
|  |  |  |  |
| anti-Nipah glycoprotein (G) (clone NVG-18) | MYBioSource | #MBS122195  Or  Ab02865-1.1 | 1:10000 |
| anti-Nipah glycoprotein (G) MAB12306 | The Native Antigen Company | MAB12306 | 1:2000 |
| anti-Nipah glycoprotein (G) 48D3 | Creative Diagnostics | CABT-NS1156 | 1:2000 |

# Supplementary Note 1

Code for logistic regression

Nip <- read.csv("NipahChalData2.csv")

Nip

# Model the data using general linear regression

NipModel <- glm(Live ~ log.FGpvN2 , data = Nip, family = binomial)

summary(NipModel)

#define new data frame that contains predictor variable

Newdata <- data.frame (log.FGpvN2 = seq (min (Nip$log.FGpvN2) -.5, max(Nip$log.FGpvN2), len=500))

#use fitted model to predict values

newdata$Live = predict(NipModel, newdata, type="response")

#plot logistic regression curve

Plot (Nip$log.FGpvN2, Nip$Live, yaxt="n", xlim=c(0.5,3), ylim=c(0,1), xlab="log F+G pseudovirus neutralization titer", ylab="Dead Live", title("Logistic Regression") )

lines(newdata$Live ~ newdata$log.FGpvN2, lwd=2)

NipahChalData2.csv

| Animal# | Vaccine | Live | Felisa | Ftitre | FGpvN2 | GpvN2 | log.F.ELISA | log.F.titre | log.FGpvN2 | log.GpvN2 |
| --- | --- | --- | --- | --- | --- | --- | --- | --- | --- | --- |
| 1 | 1 | 1 | 0.7624 | 15.7569 | 34.9 | 106.8 | -0.1178 | 1.1975 | 1.5428 | 2.0286 |
| 2 | 1 | 1 | 0.6153 | 21.9036 | 25.0 | 12.5 | -0.2109 | 1.3405 | 1.3979 | 1.0969 |
| 3 | 1 | 0 | 1.1569 | 29.1572 | 12.5 | 12.5 | 0.0633 | 1.4647 | 1.0969 | 1.0969 |
| 4 | 1 | 0 | 0.7376 | 12.5000 | 12.5 | 12.5 | -0.1322 | 1.0969 | 1.0969 | 1.0969 |
| 5 | 1 | 1 | 0.9358 | 19.1853 | 44.8 | 12.5 | -0.0288 | 1.2830 | 1.6513 | 1.0969 |
| 6 | 1 | 1 | 1.7870 | 88.1096 | 26.4 | 12.5 | 0.2521 | 1.9450 | 1.4216 | 1.0969 |
| 7 | 2 | 1 | 0.5799 | 19.9286 | 49.7 | 12.5 | -0.2367 | 1.2995 | 1.6964 | 1.0969 |
| 8 | 2 | 1 | 0.8760 | 20.9212 | 12.5 | 12.5 | -0.0575 | 1.3206 | 1.0969 | 1.0969 |
| 9 | 2 | 1 | 0.9084 | 21.3454 | 24.1 | 12.5 | -0.0417 | 1.3293 | 1.3820 | 1.0969 |
| 10 | 2 | 1 | 0.6708 | 12.5000 | 167.3 | 54.8 | -0.1734 | 1.0969 | 2.2235 | 1.7388 |
| 11 | 2 | 1 | 1.3285 | 32.0014 | 184.6 | 51.2 | 0.1234 | 1.5052 | 2.2662 | 1.7093 |
| 12 | 2 | 1 | 2.0300 | 66.3604 | 293.1 | 223.6 | 0.3075 | 1.8219 | 2.4670 | 2.3495 |
| 13 | 3 | 0 | 0.6522 | 12.5000 | 19.0 | 12.5 | -0.1856 | 1.0969 | 1.2788 | 1.0969 |
| 14 | 3 | 0 | 0.6476 | 12.5000 | 12.5 | 14.0 | -0.1887 | 1.0969 | 1.0969 | 1.1468 |
| 15 | 3 | 0 | 0.6247 | 12.5000 | 12.5 | 12.5 | -0.2043 | 1.0969 | 1.0969 | 1.0969 |
| 16 | 3 | 0 | 0.6945 | 12.5000 | 12.5 | 12.5 | -0.1584 | 1.0969 | 1.0969 | 1.0969 |
| 17 | 3 | 0 | 0.6708 | 22.0954 | 16.0 | 12.5 | -0.1734 | 1.3443 | 1.2041 | 1.0969 |
| 18 | 3 | 0 | 0.5890 | 12.5000 | 12.5 | 12.5 | -0.2299 | 1.0969 | 1.0969 | 1.0969 |
